# Transcriptomic Analysis Identified Two Subtypes of Brain Tumor Characterized by Distinct Immune Infiltration and Prognosis

**DOI:** 10.3389/fonc.2021.734407

**Published:** 2021-10-15

**Authors:** Xilin Shen, Xiaoli Wang, Hongru Shen, Mengyao Feng, Dan Wu, Yichen Yang, Yang Li, Meng Yang, Wei Ji, Wei Wang, Qiang Zhang, Fangfang Song, Ben Liu, Kexin Chen, Xiangchun Li

**Affiliations:** ^1^ Tianjin Cancer Institute, National Clinical Research Center for Cancer, Key Laboratory of Cancer Prevention and Therapy, Tianjin Medical University Cancer Institute and Hospital, Tianjin Medical University, Tianjin, China; ^2^ Department of Obstetrics and Gynecology, Tongji Hospital, Tongji Medical College, Huazhong University of Science and Technology, Wuhan, China; ^3^ Department of Epidemiology and Biostatistics, National Clinical Research Center for Cancer, Key Laboratory of Cancer Prevention and Therapy, Key Laboratory of Molecular Cancer Epidemiology of Tianjin, Tianjin Medical University Cancer Institute and Hospital, Tianjin Medical University, Tianjin, China; ^4^ Department of Maxillofacial and Otorhinolaryngology Oncology, National Clinical Research Center for Cancer, Key Laboratory of Cancer Prevention and Therapy, Tianjin Medical University Cancer Institute and Hospital, Tianjin Medical University, Tianjin, China

**Keywords:** brain tumor, immune infiltration, prognosticator, transcriptome, molecular subtype

## Abstract

**Background:**

Brain tumor ranks as the most devastating cancer type. The complex tumor immune microenvironment prevents brain tumor from receiving therapeutic benefits. The purpose of this study was to stratify brain tumors based on their distinct immune infiltration signatures to facilitate better clinical decision making and prognosis prediction.

**Methods:**

We developed a deep learning model to characterize immune infiltration from transcriptome. The developed model was applied to distill expression signatures of transcriptome of brain tumor samples. We performed molecular subtyping with the extracted expression signatures to unveil brain tumor subtypes. Computational methods, including gene set enrichment analysis, Kaplan-Meier survival and multivariate Cox regression analyses, were employed.

**Results:**

We identified two distinctive subtypes (i.e. C1/2) of brain tumor featured by distinct immune infiltration signatures. The C1 subtype is characterized by protective immune infiltration signatures, including high infiltration of CD8+ T cells and activation of *CX3CL1*. The C2 subtype has an extensive infiltration of tumor-associated macrophages and microglia, and was enriched with immune suppressive, wound-healing, and angiogenic signatures. The C1 subtype had significantly better prognosis as compared with C2 (Log-rank test, HR: 2.5, 95% CI: 2.2 – 2.7; *P* = 8.2e-78). This difference remained statistically significant (multivariate Cox model, HR: 2.2, 95% CI: 1.7 – 2.9; *P* = 3.7e-10) by taking into account age, gender, recurrent/secondary status at sampling time, tumor grade, histology, radio-chemotherapy, *IDH* mutation, *MGMT* methylation, and co-deletion of 1p and 19q. This finding was validated in six datasets. The C2 subtype of glioblastoma patients with *IDH* mutation has poor survival analogous to those without *IDH* mutation (Log-rank test, adjusted *P* = 0.8), while C1 has favorable prognosis as compared with glioblastoma of C2 subtype with *IDH* mutation (Log-rank test, adjusted *P* = 1.2e-3) or without *IDH* mutation (Log-rank test, adjusted *P* = 1.3e-6).

**Conclusions:**

We identified two distinctive subtypes of brain tumor with different immune infiltration signatures, which might be helpful as an independent prognosticator for brain tumor.

## Introduction

Brain tumors are highly aggressive and rank among the most fatal and devastating of diseases (1). The standard treatments for brain tumors include chemotherapy and radiotherapy in addition to surgical removal (2). However, the efficacy varies considerably, with some patients showing rapid resistance while others have a more durable response (3). In addition, a range of postoperative complications can occur, including seizure, loss of movement ability, visual impairment, or impairment of speech and comprehension.

Better understanding of key genomic alterations in brain tumor leads to effective treatment options for patients. For example, lack of *MGMT* methylation is associated with reduced benefit from temozolomide (4). *IDH1/IDH2* mutation and co-deletion of chromosome arms 1p and 19q are associated with radio-chemotherapy response and survival outcome (5). Meanwhile, high-throughput analyses of genomic and transcriptomic data have led to a refined classification system of brain tumor to promote effective clinical therapeutics. The WHO classification of central nervous system (CNS) introduced in 2016 defined tumor entities based on molecular characteristics in addition to traditional morphologic findings (2). Nevertheless, clinical heterogeneity remains an intractable issue. For instance, patients of astrocytoma without IDH mutation have diverse clinical outcomes (6).

Brain tumor (7, 8) microenvironment is immunologically distinct from other cancer types (7). Tumorigenesis can cause damage to the blood-brain barrier, facilitating the infiltration of immune cells from peripheral circulation into brain (7). A compromised blood-brain barrier can activate wound healing and angiogenesis, which promotes cancer progression and confers immune suppression (8, 9). Meanwhile, brain tumor is immunologically “cold” in that tumor-associated macrophages and microglia (TAMs) prevent tumor from activating the immune response (10, 11).

In this study, we established a model to distill expression signatures from the transcriptome of brain tumor tissues. We revealed two subtypes of brain tumor with distinct immune infiltration signatures: genomic alteration and prognosis. Our findings were validated in 11 previous datasets.

## Materials and Methods

### Data Collection

Brain tumor transcriptomes of 3810 patients were downloaded from the Genomic Data Commons, the NCBI Gene Expression Omnibus, the International Cancer Genome Consortium, the Chinese Glioma Genome Atlas, the European Bioinformatics Institute, and GlioVis (**Table S1**). Each dataset has more than 100 patients accompanied by their vital status and period of follow-up. Histology, tumor grade, radio-chemotherapy treatment, recurrent/secondary status at sampling time, *IDH* mutation, *MGMT* methylation, and co-deletion of 1p and 19q data were collected if available (**Table S1**). Meanwhile, we collected 93,293 single-cell RNA profiles subjected to Smart-Seq2 sequencing protocol from 16 previous studies (**Table S2**) from brain cancer, lung cancer, colorectal cancer, ovarian cancer, melanoma, and head and neck squamous cell carcinoma. These single cell datasets encompass T cells, B cells, monocytes, macrophages, natural killer cells, dendritic cells, cancer cells, and other nonmalignant cells including fibroblast, epithelia cells, gliocytes, and neurons. In addition, we manually curated a list of genes related to tumor microenvironment, immune cells, immune checkpoint blockade therapy response, and prognosis (**Table S3**).

### Data Preprocessing

For each single-cell dataset, we performed logarithmic transformation as log_2_(TPM/10 + 1). We clipped gene expression values at 99% quantile values of all genes. Subsequently, we employed R package *preprocessCore* (version 1.40.0) (12) to perform quantile normalization. *We applied ComBat* routine implemented in R package *sva* (version 3.26.0) (13) to perform batch effect correction for the normalized expression data of bulk brain tumors.

### Feature Representation Learning Of Single-Cell Transcriptome

We developed a feature encoder through self-supervised feature representation learning. The feature encoder could learn nonlinear feature representations of transcriptomes in a reduced dimensional space.

The feature encoder was trained with a self-supervised deep learning algorithm based on contrastive learning (14). Specifically, contrastive learning allows the feature encoder to learn representations in a label-free manner (**Figure S1**). Positive pairs are defined as two different noise-adding views of the same transcriptome (*V_q_*, *V_k+_*). Two different transcriptomes form a negative pair (*V_q_*, *V_k-_*). *V_k-_* came from a dictionary of transcriptomes {V_k1-_, V_k2-_,…, V_n1-_}, which was defined on-the-fly by a set of trained data. Contrastive loss aims to minimize the distance between the positive pair and maximize the distance between the negative pair. The function of contrastive loss is defined as:


$$L_{V_{\text{q}},V_{k+},\{V_{k-}\}}=-\log\frac{\exp(V_{q}\times V_{k+}/\tau )}{\exp(Vq\times Vk+/\tau )+\sum_{k-}\exp(V_{q}\times V_{k-}/\tau )}$$

where τ is a temperature hyper-parameter (15). In this analysis, the similarity of each pair was calculated based on the expression features extracted from the feature encoder. In this manner, the feature encoder was driven to learn features of transcriptomes by contrastive loss.

### Network Architecture and Training

In our task, the feature encoder was trained to learn the same representation of different noise-adding views of the same single-cell transcriptome and dissimilar representation of different cells.

The 93,293 single cells were randomly divided into a training set (*N* = 83,964) and a validation set (*N* = 9,329). We logarithmically transformed the transcriptomes of the preselected genes (**Table S3**) and scaled to a range of 0 to 1 before feeding them into the feature encoder. At each epoch, we made noise through random zero out and shuffling and added Gaussian noise (mean: 0, standard deviation: 0.1) to 20% of genes for all transcriptomes.

The feature encoder was an 18 layered ResNet (16). We replaced the convolutional layer in the original ResNet with a linear layer to allow it to process gene expression data. For each residual block, the input skips training from a few layers and is connected directly to the output. Moreover, we set the project head with 128 output neurons. The use of multi-layer perceptron (MLP) as project head was demonstrated to be beneficial for contrastive learning. The architecture of the feature encoder was provided as **Figure S2**.

We employed stochastic gradient descent algorithm (17) as the optimizer. The weight decay of the optimizer is 1e-4 and the momentum is 0.9. We set batch size for each training iteration of 256. The initial learning rate was 0.03 and decay with a cosine annealing schedule. We set the contrastive learning dictionary size to 3072. The momentum and τ of contrastive loss were set to 0.999 and 0.2, respectively. The model was trained in parallel on two graphic processing units for 300 epochs. The model was developed with *PyTorch* (v1.3.0) package.

### Molecular Subtyping of Brain Tumor

The developed feature encoder was applied to extract expression signatures from bulk sample transcriptomes. Specifically, we extracted feature representations from the developed feature encoder applied to the expression data of TCGA pan-cancer. The feature encoder transformed the expression profile of each bulk sample into 128 features, which was determined by the output neurons of feature encoder. The extracted features were hierarchically clustered through R package *ConsensusClusterPlus* (version 1.42.0) (18). The obtained clusters were further grouped into expression signatures because of the high negative correlations among these clusters (**Figure S3**). Subsequently, we dichotomized brain tumor patients based on each expression signature and selected one that can better represent unique immune infiltration signature of brain tumor. Specifically, we used *R* package *Ckmeans.1d.dp* (version 4.2.1) (19) to perform k-means clustering. The k-means clustering cutoff value closest to the median value of signature was selected as the optimal cutoff to dichotomize samples. We used R package *fgsea* (version 1.6.1) (20) to perform gene set enrichment analysis (GSEA) for a gene set related to unique immune infiltration properties of brain tumor such as microglia and reactive gliosis (**Table S4**). We kept the signature that ranked on the top to dichotomize patients as mentioned above for downstream analysis. A flowchart illustrating this procedure was provided in **Figure S3**.

### Linear Feature Encoder Comparison

To examine the advantage of self-supervised learning paradigm, we employed principle component analysis (PCA) as a linear feature encoder and compared the PCA features with the deep learning features. Specifically, we performed PCA on single cell transcriptomes of the 2616 filtered genes with python package *sklearn* (v0.24.1). The principle components of single cells were then projected to brain tumor transcriptome. Then, brain tumor patients were dichotomized through hierarchical clustering based on R package *ConsensusClusterPlus* (version 1.42.0) (18).

### Association Between Molecular Subtypes and Clinical Data

We analyzed the association between molecular subtypes with immune and genomic alteration signatures, which include immune cellular fractions, immunomodulatory expressions, oncogenic and immune pathways, genomic alterations, driver mutations, and molecular subtypes of glioblastoma proposed by the Cancer Genome Atlas (TCGA) (21). We used CIBERSORT to estimate the proportions of 22 immune cell types based on LM22 matrix (22). We performed paired t-test for 78 genes related to immunomodulation (23) in the 11 collected datasets. In addition, we performed GSEA based on R pakage *fgsea* (version 1.6.1) (16) for cancer hallmark (24) and immune-related gene sets (19) (**Table S4**). Continuous variables were evaluated by Wilcoxon rank sum test, while discrete variables were evaluated *via* Chi-square test if not specified. For GSEA, *P*-values were calculated based on 10,000 permutations. Kaplan-Meier survival analysis and multivariate Cox hazards model were utilized to analyze the association of subtypes and prognosis, which were carried out with R *survival* package (2.40-3).

### Statistical Analysis

As described above, we generally employed Wilcoxon rank sum test or Chi-squared test for the statistical analysis as appropriated, if unspecified. The paired t-test was used for the analysis of immunomodulators. The *P* value of enrichment analysis were calculated based on 10,000 permutations. We employed Kaplan-Meier analysis to estimate survival distribution. Cox proportional-hazards model was utilized for multi-variable survival analysis. We applied log-rank test to compare the statistic difference of survival curves between two groups. All figures and statistical analysis were conducted using R software (version 3.6.1). A *P* < 0.05 was considered as statistically significant. All statistical tests were two-sided. *P*-values were adjusted with FDR method.

## Results

### Patients and Analytic Pipeline

RNA profiles and clinical data of 3810 brain tumor patients were collected from 11 public studies. The baseline characteristics are shown in **Table S5**. Glioma and medulloblastoma account for 83% (3146) and 16% (624), respectively. Among 2951 patients with cancer type information, the primary, recurrent, secondary, and post-treatment tumor account for 71% (2105), 11% (332), 1% (40), and 16% (474), respectively. In the glioma cohort (3146), patients with tumor grade II, III, and IV respectively accounted for 24% (743), 28% (872), and 39% (1226); 10% (305) of patients did not have tumor grade information. Meanwhile, there were 2960 glioma patients with pathological information. This glioma cohort consisted of diverse pathological subtypes such as astrocytoma (27%), oligodendroglioma (21%), oligoastrocytoma (8%), and glioblastoma (44%). Among 2289 glioma patients with *IDH* mutation examined, 55% of them (1254) had *IDH* mutation. Among 1098 of these 1254 patients with co-deletion of 1p/19q tested, 42% (459) carried co-deletion of 1p and 19q. Among 1731 patients tested for *MGMT* methylation, 59% (1028) of them were positive for hypermethylation of *MGMT* promoter. Among 1226 patients with radio-chemotherapy treatment information, the proportion of patients treated with chemotherapy, radiotherapy, and a combination of both were 15% (184), 23% (281), and 62% (761), respectively.

**Figure 1:**
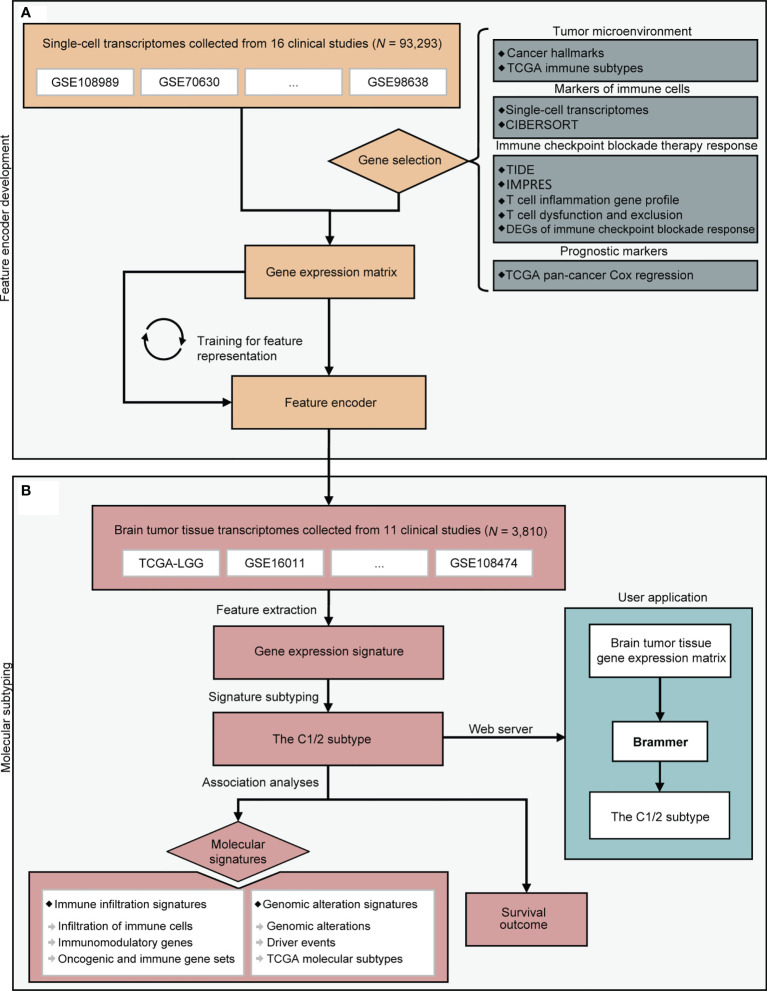
A flowchart depicting all procedures conducted in this study. The upper panel **(A)** describes the steps involved in the development of a deep learning model to learn feature representation from single-cell transcriptomes. The lower panel **(B)** depicts molecular subtyping of brain tumors and downstream analysis tasks. CIBERSORT, TIDE, and IMPRES were referenced from (22, 25, 26). DEG, differentially expressed gene; TCGA, the Cancer Genome Atlas.

A flowchart depicting the whole procedures of this study is shown in **Figure 1**. We collected 93,293 single-cell RNA profiles from 16 published datasets. A total of 2,616 genes were selected for the analysis. These genes were associated with tumor microenvironment, immune cells, immune checkpoint blockade therapy response, and prognosis (See *Methods*). We developed a self-supervised deep learning model based on single-cell RNA profiles of these 2616 genes to decipher gene expression signatures from transcriptomes. Subsequently, we applied this developed feature encoder to extract expression signatures from transcriptome of bulk brain tumor samples (See *Methods* and **Figure S3**). We then examined the association of expression signature with immune signatures, genomic alteration, and prognosis.

### Differences of Immune Infiltration Signatures in C1 *Versus* C2 Subtype

**Figure 2:**
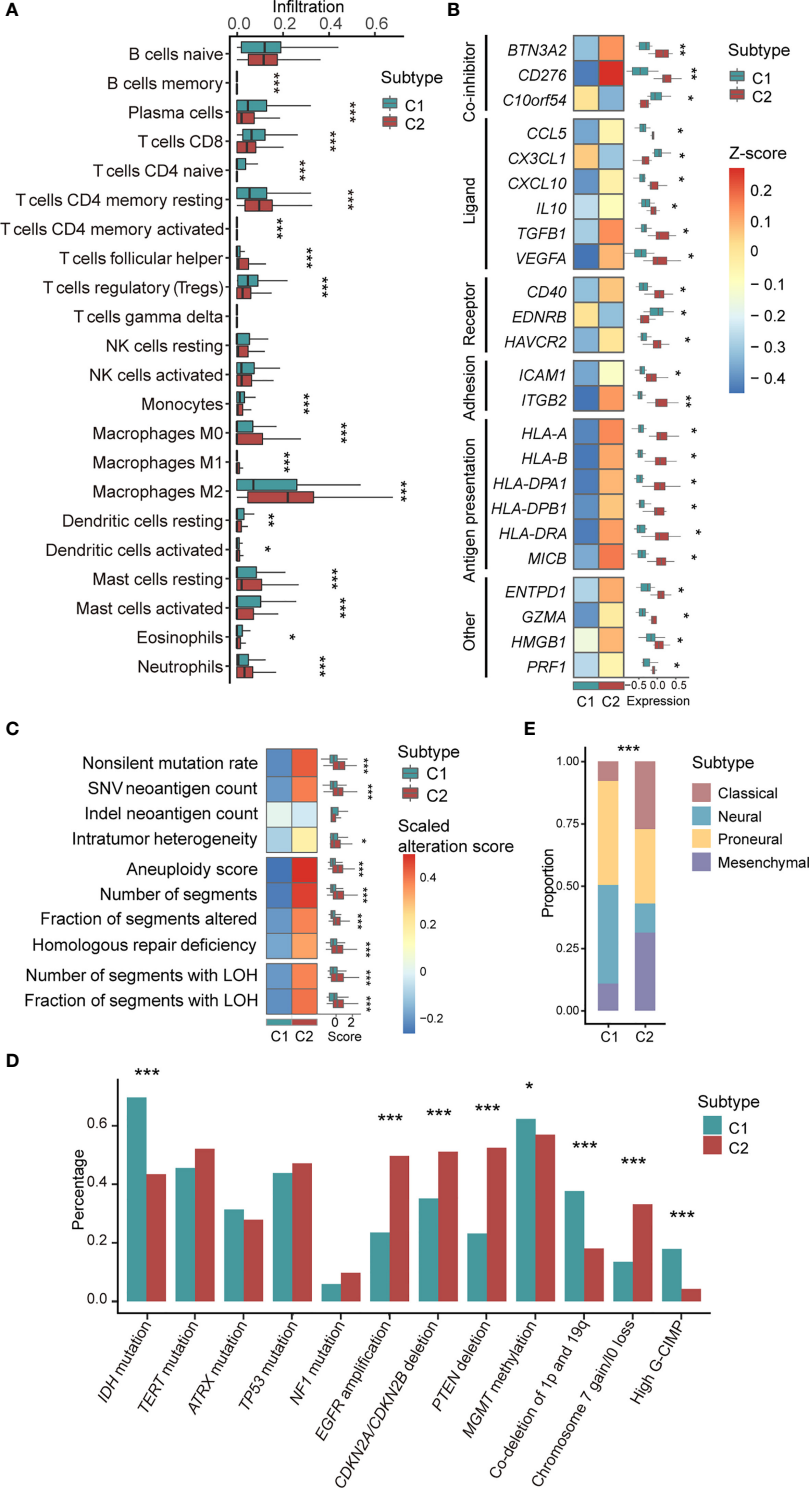
Association between C1/2 subtypes with genomic and transcriptomic signatures. **(A)** The proportion of infiltrated immune cell types in C1 *versus* C2 subtype. **(B)** The median expression levels of immunomodulatory genes across 11 brain tumor datasets in C1 *versus* C2 subtype. **(C)** Genomic alteration signatures in C1 *versus* C2 subtype in TCGA low-grade glioma cohort. **(D)** Alteration prevalence of driver events in C1 *versus* C2 subtype. **(E)** The proportion of TCGA molecular subtypes in C1 *versus* C2 subtype. *P* values were subjected to multiple hypothesis correction. *P < 0.05, **P < 0.01, ***P < 0.001. LOH, loss of heterozygosity; G-CIMP, CpG island methylation phenotype.

The results obtained from CIBERSORT (22) showed that 18 of 22 types of immune cells were significantly different between C1/2 subtypes (**Figure 2A**; Wilcoxon rank sum test, *P* < 0.05). All types of TAMs (i.e. M0, M1, M2), CD4+ follicular helper T cells, and neutrophils had higher infiltration rate in C2 as compared with C1 subtype (**Figure 2A**). Contrastively, C1 had higher infiltration of CD8+ T cells, plasma cells, and dendritic cells than C2 subtype (**Figure 2A**). The infiltration of the other cell types was provided in **Table S6**. Furthermore, the immune infiltration of C1 *versus* C2 exhibited consistent trends among different brain tumor subtypes (**Figure S4**). For example, there are higher infiltration of plasma cells and lower infiltration of M1 and M2 macrophages in C1 subtype (**Figure S4**).

We observed that 24 immunomodulatory genes were differentially expressed in C1 *versus* C2 subtype (**Figure 2B**; Paired t-test, *P <*0.05). Specifically, *C10orf54*, *CX3CL1*, and *EDNRB* were highly expressed in C1 *versus* C2 subtype (**Figure 2B**). *CD276*, *CCL5*, *CXCL10*, and *HMGB1* and the other 17 immunomodulatory genes were significantly upregulated in C2 *versus* C1 subtype (**Figure 2B**). The detailed expression of all immunomodulatory genes were provided in **Table S7**.

Enrichment analysis of 50 cancer hallmarks and 132 immune signaling modules showed that CSF-1, MYC, TGF-β, JAK/STAT3, IFN-α, and the other 29 signaling pathways were enriched in C2 *versus* C1 subtype (**Figure S5**, *P* < 0.05). For 11 signaling modules, including core serum response, proliferation, DNA repair, and E2F target pathways, the same trends were validated across brain tumor subtypes (**Figure S5**, *P* < 0.05).

### C1/2 Subtypes Were Significantly Associated With Genomic Alterations

In the TCGA low-grade glioma, non-silent mutation burden, intratumor heterogeneity, aneuploidy, and the other six types of genomic variation were significantly higher in C2 *versus* C1 subtype (**Figure 2C** and **Table S8**; Wilcoxon rank sum test, *P* < 0.05). The corresponding trends were observed in astrocytoma and oliodendroglioma patients (**Figure S6**). In the TCGA glioblastoma cohort, there was no difference among the aforementioned variations except for segments of copy number variation (**Figure S6**).

We also examined the association of C1/2 subtypes and driver gene mutations of brain tumors that linked to prognosis and therapeutic resistance (**Table S6**). Our finding showed that four driver events were significantly higher in C1 *versus* C2 subtype, including *IDH* mutation, hypermethylation of *MGMT* promoter, high CpG island methylation phenotype (G-CIMP), and co-deletion of 1p and 19q (**Figure 2D**; Chi-squared test, *P* < 0.05). Four driver events were significantly higher in C2 *versus* C1 subtype such as *EGFR* amplification, deletion of *CDKN2A*/*CDKN2B* and *PTEN*, gain of chromosome 7, and/or loss of chromosome 10 (**Figure 2D**; Chi-squared test, *P* < 0.05). Across brain tumor subtypes, such as glioblastoma and low-grade glioma, differences in mutation rates showed the same trend in eight driver events among C1/2 subtypes, including *CDKN2A/CDKN2B* and *PTEN* deletion, *IDH* mutation, and co-deletion of 1p and 19q (**Figure S7**).

In addition, we found that C1/2 subtypes were linked to TCGA molecular subtypes, namely classical, neural, proneural, and mesenchymal subtypes (21) (**Figure 2E**). Neural [168(37%) *versus* 104(11%); Chi-squared test, *P* = 6.2e-29] and proneural subtypes [186(41%) *versus* 243(26%); Chi-squared test, *P* = 6.2e-29] were significantly enriched in C1 *versus* C2 subtype. C2 had higher proportions of classical [307(33%) *versus* 56(12%); Chi-squared test, *P* = 1.4e-16] and mesenchymal subtypes [265(29%) *versus* 44(10%); Chi-squared test, *P* = 2.3e-15] as compared with C1 subtype.

### C1/2 Subtypes Were Significantly Associated With Clinical Characteristics

Clinical characteristics of brain tumor patients were provided in **Table S9**. C2 subtype had lower Karnofsky scores (Median: 80 *vs.* 90, Wilcoxon rank sum test, *P* = 3.4e-6) and higher tumor microvascular infiltration rate *versus* C1 subtype (61/76, 80% *vs.* 31/65, 48%; OR: 4.2, 95% CI: 2.0 – 8.7; Chi-squared test, *P* = 1.8e-4). Among patients with recurrence, C1 subtype had marginally significant lower distant recurrence rate (4/23, 17% *vs.* 19/48, 40%; OR: 0.3, 95% CI: 0.1 – 1.1) and higher local recurrence rate (19/23, 83% *vs.* 29/48, 60%; OR: 3.1, 95% CI: 0.9 – 10.6) as compared with C2 subtype (Chi-squared test, *P* = 0.1). There were no significant differences in family history of cancer, pre-diagnostic symptoms, and tumor location between C1/2 subtypes (Chi-squared test, *P* > 0.5).

Kaplan-Meier survival analysis showed that C1 subtype has better survivability than C2 subtype (**Figure S8A**; Log-rank test, *P* = 8.2e-78) in the combined cohort of 3810 patients. This result was also observed in each individual in the 11 datasets (**Figure S8A**; Log-rank test, *P* < 0.05). Moreover, the difference remained significant in the combined cohort after controlling for confounding factors such as age, gender, tumor, histology, radio-chemotherapy, recurrent/secondary status, *IDH* mutation status, *MGMT* methylation status, and co-deletion of 1p and 19q (**Figures S8B**, **S12**; Multivariate Cox model, HR: 2.2, 95% CI: 1.7 – 2.9; Log-rank test, *P* = 3.7e-10). The independent association of C1/2 subtypes with prognosis from the multivariate model remained significant in six individual datasets and exhibited the same trend in the other four datasets (**Figures S8B**, **S12**; Log-rank test, *P* < 0.05). In the TCGA glioma cohort, surgery type was taken into consideration additively. In the medulloblastoma cohort (i.e. GSE85217), clinically relevant confounding factors, such as age, gender, and molecular subtypes, were included.

We observed that C1/2 subtypes of PCA have significantly different overall survival in seven independent datasets (**Figure S9**; Log-rank test, *P* < 0.05). Cox analysis (**Table S10**) shows that C1/2 subtypes have prognosis significance in five individual datasets (Log-rank test, *P* < 0.05) and did not show any trend in three datasets (i.e. E-MTAB-3892, TCGA-GBM, GSE13041). In summary, the association between prognosis and expression signatures derived from deep learning is more general as compared with PCA.

### C1/2 Subtypes Have Prognostic Significance Across Brain Tumor Subtypes

**Figure 3:**
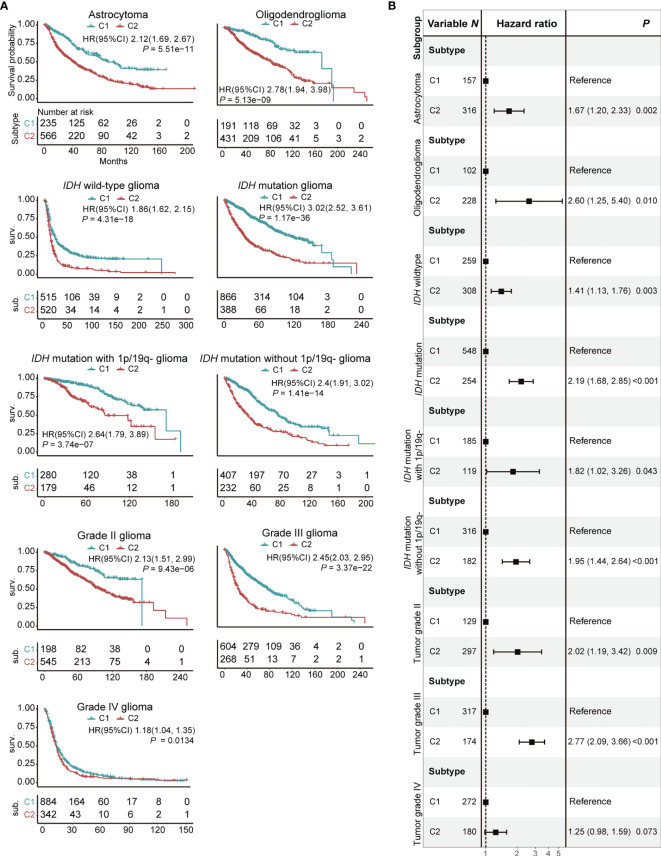
Prognostic significance of C1/2 subtypes stratified by different clinical variables. **(A)** Kaplan-Meier survival analysis of C1 *versus* C2 subtype. **(B)** Combined forest plot portraying multivariate Cox regression analysis of C1/2 subtypes after controlling age, gender, histology, *IDH* mutation, *MGMT* methylation, and co-deletion of 1p and 19q. 1p/19q-, co-deletion of 1p and 19q; HR, hazard ratio; CI, confidence interval.

We examined the association between C1/2 subtypes and prognosis of glioma patients with respect to histology, genomic alteration, and grade. The glioma patients were divided into nine subgroups: astrocytoma, oligodendroglioma, glioma with or without *IDH* mutation, glioma with *IDH* mutation with or without co-deletion of 1p and 19q, tumor grade II, III, and IV (**Figure 3A**). The C2 subtype has significantly poorer survival outcome than C1 in all subgroups (**Figure 3A**; Log-rank test, *P* < 0.05). In addition, the difference remained significant in eight out of these nine subgroups and marginally significant in grade IV glioma after taking into account age, gender, histology, *IDH* mutation status, *MGMT* methylation status, and co-deletion of 1p and 19q (**Figures 3B**, **S12**; Log-rank test, *P* < 0.05). The dataset was taken as strata variable in multivariate Cox model.

**Figure 4:**
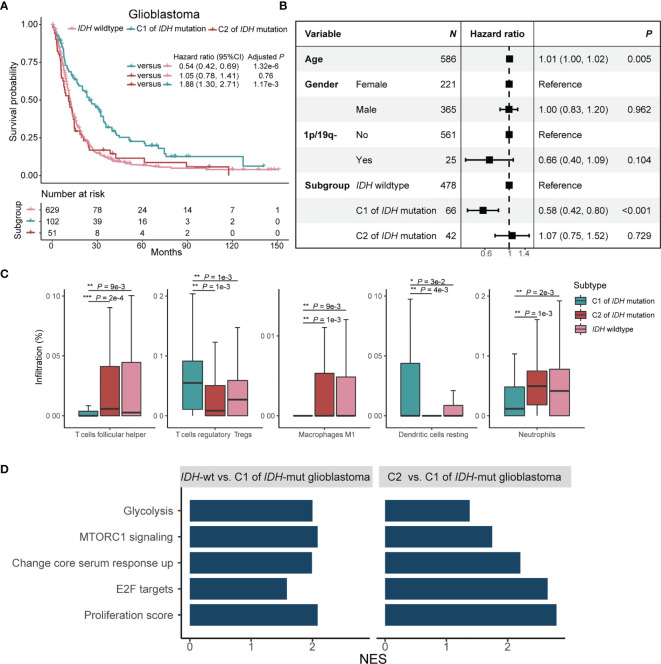
Prognostic significance and immune signatures of *IDH* mutation plus C1/2 subtype in glioblastoma patients. **(A)** Kaplan-Meier survival analysis of glioblastoma in patients without *IDH* mutation, C1 subtype with *IDH* mutation, and C2 subtype with *IDH* mutation. **(B)** Multivariate Cox regression analysis of C1/2 subtypes by ruling out confounding impacts such as age, gender, and co-deletion of 1p and 19q. 1p/19q-, co-deletion of 1p and 19q; CI, confidence interval. **(C)** Immune cell infiltration rates of the trichotomy of glioblastoma. **(D)** Biological pathway enrichment scores of glioblastoma without *IDH* mutation and C2 subtype of glioblastoma with *IDH* mutation relative to C1 subtype of glioblastoma with *IDH* mutation. NES, normalized enrichment score. NES, number of enrichment score.

The C2 subtype of glioblastoma with *IDH* mutation has poor survival outcome analogous to glioblastoma without *IDH* mutation (**Figure 4A**; Log-rank test, *P* = 0.8). The C1 subtype of glioblastoma with *IDH* mutation, meanwhile, has a favorable survival outcome *versus* C2 subtype (Log-rank test, *P* = 1.2e-3) or glioblastoma without *IDH* mutation (Log-rank test, *P* = 1.3e-6). The result remained significant after ruling out confounding impacts of age, gender, and co-deletion of 1p and 19q (**Figure 4B**). CIBERSORT analysis demonstrated that there are high infiltration rates of regulatory T cells and dendritic cells and low infiltration rates of follicular helper T cells, M1 macrophages, and neutrophils in C1 subtype of glioblastoma with *IDH* mutation (**Figure 4C**; Wilcoxon rank sum test, *P* < 0.05). GSEA analysis showed that glycolysis, MTORC1, core serum response, proliferation, and E2F signaling pathways were enriched in the C2 subtype of glioblastoma with *IDH* mutation and *IDH* wildtype glioblastoma as compared with C1 subtype of glioblastoma with *IDH* mutation (**Figure 4D**; *P* < 0.05).

We further stratified glioblastoma patients through different treatment modalities. For patients with radiotherapy (*N* = 86), Kaplan-Meier survival analysis showed that C2 subtype has better overall survival than C1 subtype (**Figure S10**, Log-rank test, *P* = 3.3e-2). The same trend was also observed in C1/2 subgroup of patients with radio-chemotherapy (*N* = 49, **Figure S10**, Log-rank test, *P* = 0.2).

Kaplan-Meier survival analysis showed that C2 subtype had worse progression-free survival as compared with C1 subtype in TCGA glioma cohort (**Figure S11**, Log-rank test, *P* = 6.1e-4). The difference remained significant in radio-chemotherapy patients (**Figure S11**, Log-rank test, *P* = 5.3e-3) and showed the same trend in radiotherapy alone patients (**Figure S11**, Log-rank test, *P* = 0.4). Progression-free survival was not analyzed for the chemotherapy group due to the limited sample size (**Table S11**).

## Discussion

The immune microenvironment plays pivotal roles in cancer progression of brain tumor (27). It is of importance to mine potential heterogeneity of immune infiltration for better guidance of treatment. This study represents an attempt to identify new subtypes of brain tumor based on immune infiltration signature. It extends the previous classification systems that are mainly defined on histology and genome (2). The importance of identifying the C1/2 subtypes lies in their markedly different survival outcomes due to their distinct immune infiltration. Our findings will facilitate the elucidation of distinct immune infiltration in the development and prognosis prediction of brain tumors.

We trained the feature encoder with nearly 100,000 transcriptomes from multiple cancer types. The large amount of data were applied to address the big data requirement of deep learning models (28) and to learn shared immune signatures across immune microenvironment. The feature encoder derived from self-supervised learning is akin to PCA in that both of them can extract representation features in a label-free manner. However, the deep neural network is able to capture the non-linear feature in contrast to linear feature reduction of PCA. The non-linear feature modeling capability of deep neural network may be better in distilling immune infiltration signatures and provide new insights as compared with PCA (**Figure S9** **)**. This was demonstrated by the identification of C1/2 subtypes that can better serve as an independent prognosticator compared with subtypes obtained from PCA.

The C1/2 subtypes can distinguish glioma patients with different prognosis stratified by histology, tumor grade, and genomic alteration. In addition, the C1/2 subtypes can also reflect differences in microvascular infiltration, distant metastasis, and radio-chemotherapy response of patients. The intrinsic distinctiveness in immunity may explain the different prognosis of C1/2 subtypes. The C1 subtype was enriched for a constellation of protective markers for prognosis such as high infiltration of CD8+ T cells, plasma cells, dendritic cells, and activation of *CX3CL1*. CD8+ T cells are the main force in maintaining anti-tumor immune responses (29). *CX3CL1* can inhibit the migration of tumor cells (30). Protective genomic alteration events, including *IDH* mutations and CpG island methylation, also occured frequently in C1 subtype. The *IDH* mutation causes aberrant methylation of DNA and histone (31) to force the appearance of CpG island methylation phenotype in glioma, both of which are favorable prognosticators in brain tumor (32).

The C2 subtype was characterized by enrichment of immune infiltration signatures. A striking characteristic of C2 subtype is the extensive infiltration of TAMs. TAM functions in immunosuppression to promote the development of a “cold” microenvironment for brain tumor (33). TAMs recruitment signatures, such as upregulated CSF-1 response circuits and highly expressed macrophage chemokine genes including *CCL5* and *VEGF* (33), were also enriched in C2 subtype. Besides, abundant signatures related to immune suppression, wound healing, and angiogenesis were detected in C2 subtype. For example, C2 subtype was poorly infiltrated with CD8+ T cells and enriched for immune suppression genes such as *IL-10*, *TGF-β*, *HAVCR2*, and *ENTPD1* (33, 34). Wound-healing programs including core serum response and JAK/STAT3 circuit were overrepresented in C2. Angiogenic signatures, including high expressions of *VEGFA* and *CD276* and amplification of *EGFR* (35–37), were abundant in C2 subtype. Aberrant tumor angiogenesis contributes to immunosuppression and tumorigenesis through subvert effector CD8+ T cells and promotes regular CD4+ T cells infiltration (38). CD276 is a putative target for CAR T-cell therapy of pediatric glioma (36). Apart from the upregulation of E2F, MYC, and G2M circuits, C2 subtype was enriched for deletion of *CDKN2A*/*CDKN2B* and *PTEN*. C2 subtype was highly infiltrated by neutrophil. Neutrophil has been reported to be associated with acquired resistance to radio-chemotherapy in brain tumor (39). Moreover, the immune infiltration patters of C1/2 subtypes were largely consistent across different tumor subtypes. Hence, the C1/2 subtypes are anticipated to be broadly implicated in brain tumor.

It is generally accepted that glioblastoma with *IDH* mutation has better prognosis than those without (40). The C2 subtype of glioblastoma with *IDH* mutation has poor survival outcomes analogous to those without *IDH* mutation, whereas the C1 subtype of glioblastoma with *IDH* mutation has significantly better survival outcomes (**Figures 4A, B**). The similar infaust prognosis of C2 subtype of glioblastoma with *IDH* mutation and glioblastoma without *IDH* mutation can be partially interpreted by the commonalities of immune infiltration status. Follicular helper T cells, M1 macrophages, and neutrophils were more enriched in them, and were associated with tumor enhancement and drug resistance (27). Furthermore, canonical pro-tumorigenic signaling pathways including E2F and MTORC1 pathways were both upregulated in C2 subtype of glioblastoma with *IDH* mutation and glioblastoma without *IDH* mutation. The C1/2 subtypes proposed in our study may improve the current glioblastoma classification system based on *IDH* mutation status to more accurately reflect prognostic discrepancy among glioblastoma patients.

Our analysis has several limitations. First, the limited availability of clinical information restricts the association analysis between C1/2 subtype with therapy response. The association between therapy outcome and progression-free survival can only be explored in TCGA glioma cohort. We cannot examine the connection between C1/2 subtypes and chemotherapy due to the limited sample size (*N* = 9). A differential trend in progression-free survival of radiation-alone patients (*N* = 21) was observed between C1/2 subtypes, and further studies should include more patients to demonstrate this difference. Second, the immune infiltration differences between C1/2 subtypes and their relationship with prognosis are still preliminary. The detailed mechanisms are still unclear and require further study.

In summary, we revealed two molecular subtypes (i.e. C1/2) of brain tumor featured by distinct immune infiltration signatures and prognosis. Our finding is helpful for better understanding of brain tumor and has potential clinical utilities.

## Data Availability Statement

We presented an R package Brammer (https://github.com/xilinshen/brammer) that can identify C1/2 subtypes based on expression matrix. The original contributions presented in the study are included in the article/**Supplementary Material**. Further inquiries can be directed to the corresponding authors.

## Author Contributions

XL and KC designed and supervised the study. XL and XS wrote the manuscript. XL, KC, and XS revised the manuscript. XL and XS analyzed the data. XS, XW, HS, MF, DW, YY, YL, and MY collected data. XL, KC, XS, WJ, WW, QZ, FS, and BL interpreted the results. All authors contributed to the article and approved the submitted version.

## Funding

This work was supported by the National Natural Science Foundation of China [31801117], the Program for Changjiang Scholars and Innovative Research Team in University in China [IRT_14R40], the Tianjin Science and Technology Committee Foundation [17JCYBJC25300], the Chinese National Key Research and Development Project [2018YFC1315600], and the Tianjin Municipal Health Commission Foundation [RC20027].

## Conflict of Interest

The authors declare that the research was conducted in the absence of any commercial or financial relationships that could be construed as a potential conflict of interest.

## Publisher’s Note

All claims expressed in this article are solely those of the authors and do not necessarily represent those of their affiliated organizations, or those of the publisher, the editors and the reviewers. Any product that may be evaluated in this article, or claim that may be made by its manufacturer, is not guaranteed or endorsed by the publisher.

## Supplementary Material

Supplementary Figure 1 A perspective of contrastive learning. V_0_, $V_{0}^{’}$ are two different views of the same sample X_0_. The feature encoder represents V_0_ and $V_{0}^{’}$ in a reduced dimensional space as R_0_ and $R_{0}^{’}$ . Contrastive learning algorithm trains the feature encoder by driving the maximum similarity between R_0_ and $R_{0}^{’}$ .

Supplementary Figure 2 The architecture of feature encoder. The feature encoder is a deep residual network of 18 layers with a multilayer perceptron project head of 128 output neurons.

Supplementary Figure 3 A flowchart depicting molecular subtyping of brain tumor. **(A)** The procedure to dichotomize brain tumor with extracted expression signatures. **(B)** Extracted signature grouping. **(C)** Signature ranking and selection. Cor, Pearson correlation coefficient; GSEA, gene set enrichment analysis; *P*-adj, adjusted *P* value.

Supplementary Figure 4 The proportion of infiltrated immune cell types in C1 *versus* C2 subtype across medulloblastoma, glioblastoma, low-grade glioma, astrocytoma, and oligodendroglioma.

Supplementary Figure 5 Significantly enriched pathways in C2 relative to C1 subtype for each brain tumor subtype.

Supplementary Figure 6 Genomic alteration signatures in C1 *versus* C2 subtype in glioblastoma, oligodendroglioma, and astrocytoma cohort. Oligo, oligodendroglioma.

Supplementary Figure 7 Alteration prevalence of driver events in C1 *versus* C2 subtype in brain tumor subtypes.

Supplementary Figure 8 Prognostic significance of C1/2 subtypes stratified by different clinical variables. **(A)** Kaplan-Meier survival analysis of C1 *versus* C2 subtype. **(B)** Combined forest plot portraying multivariate Cox regression analysis of C1/2 subtypes after controlling age, gender, histology, *IDH* mutation, *MGMT* methylation, and co-deletion of 1p and 19q. 1p/19q-, co-deletion of 1p and 19q; HR, hazard ratio; CI, confidence interval.

Supplementary Figure 9 Kaplan-Meier survival analysis of C1/2 subtypes classified by PCA.

Supplementary Figure 10 Kaplan-Meier survival analysis of C1/2 subtypes of glioblastoma with different treatment modalities.

Supplementary Figure 11 Kaplan-Meier analysis of progression-free survival differences of C1/2 subtypes in TCGA glioma cohort.

Supplementary Figure 12 Forest plot representation of multivariate Cox regression analyses in the combined, individual cohort, and different subgroups.
