# Supplementary material for: Transcriptomic Analysis Identified Two Subtypes of Brain Tumor Characterized by Distinct Immune Infiltration and Prognosis
Source: Front Oncol. 2021 Oct 15;11:734407. doi: 10.3389/fonc.2021.734407 (PMC8554158; doi:10.3389/fonc.2021.734407)
Supplement: Supplementary Figure 1 — A perspective of contrastive learning. V0, V0’ are two different views of the same sample X0. The feature encoder represents V0 and V0’ in a reduced dimensional space as R0 and R0’ . Contrastive learning algorithm trains the feature encoder by driving the maximum similarity between R0 and R0’ . [file DataSheet_1.zip › Supplementary material/Figure S10.pdf]

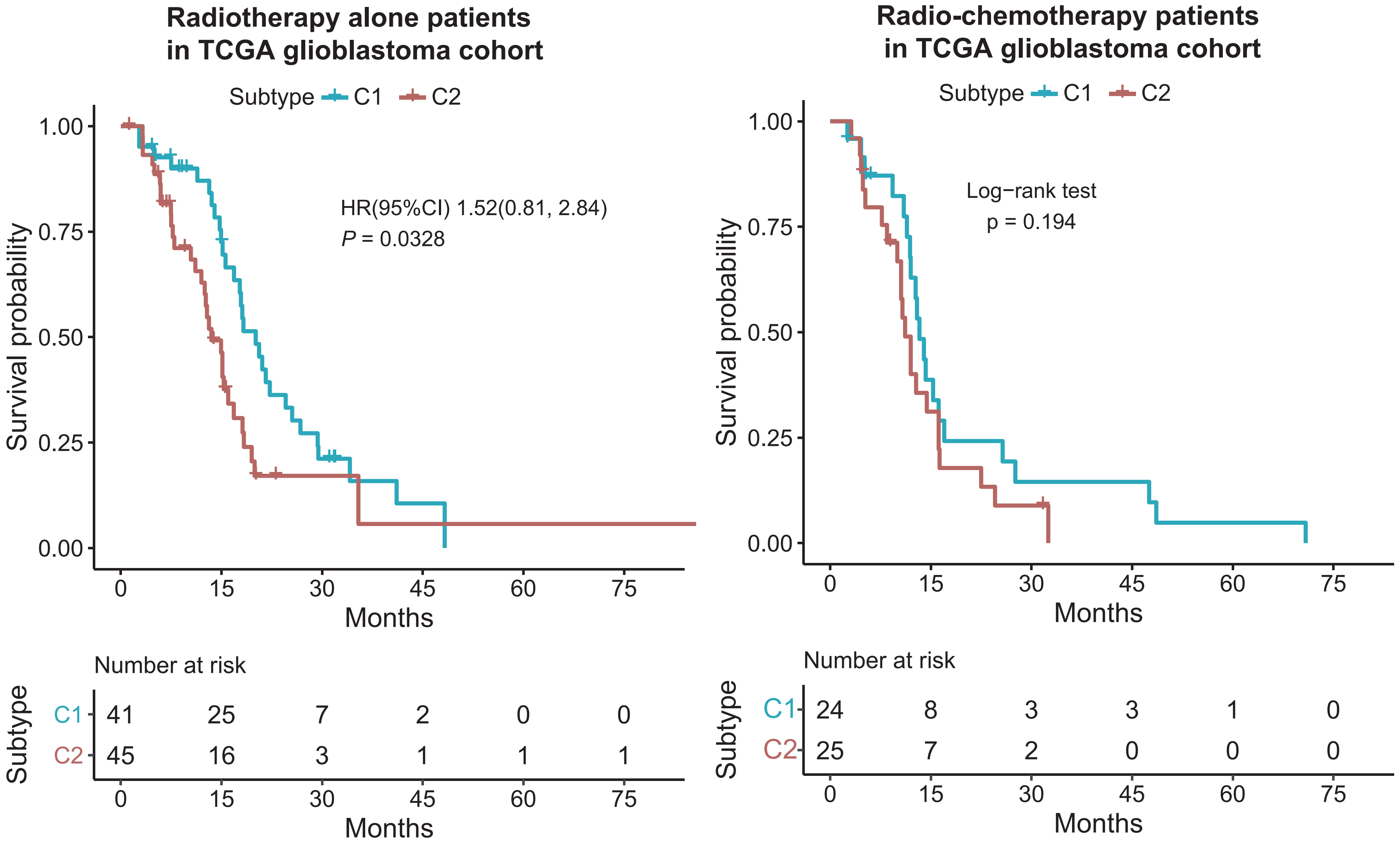
Radiotherapy alone patients
in TCGA glioblastoma cohort
Subtype C1 C2
HR(95%CI) 1.52(0.81, 2.84)
P = 0.0328
Survival probability
1.00
0.75
0.50
0.25
0.00
0 15 30 45 60 75
Months
Number at risk
Subtype
C1 41 25 7 2 0 0
C2 45 16 3 1 1 1
0 15 30 45 60 75
Months
Radio-chemotherapy patients
in TCGA glioblastoma cohort
Subtype C1 C2
Log−rank test
p = 0.194
Survival probability
1.00
0.75
0.50
0.25
0.00
0 15 30 45 60 75
Months
Number at risk
Subtype
C1 24 8 3 3 1 0
C2 25 7 2 0 0 0
0 15 30 45 60 75
Months
