# Supplementary material for: Transcriptomic Analysis Identified Two Subtypes of Brain Tumor Characterized by Distinct Immune Infiltration and Prognosis
Source: Front Oncol. 2021 Oct 15;11:734407. doi: 10.3389/fonc.2021.734407 (PMC8554158; doi:10.3389/fonc.2021.734407)
Supplement: Supplementary Figure 1 — A perspective of contrastive learning. V0, V0’ are two different views of the same sample X0. The feature encoder represents V0 and V0’ in a reduced dimensional space as R0 and R0’ . Contrastive learning algorithm trains the feature encoder by driving the maximum similarity between R0 and R0’ . [file DataSheet_1.zip › Supplementary material/Figure S11.pdf]

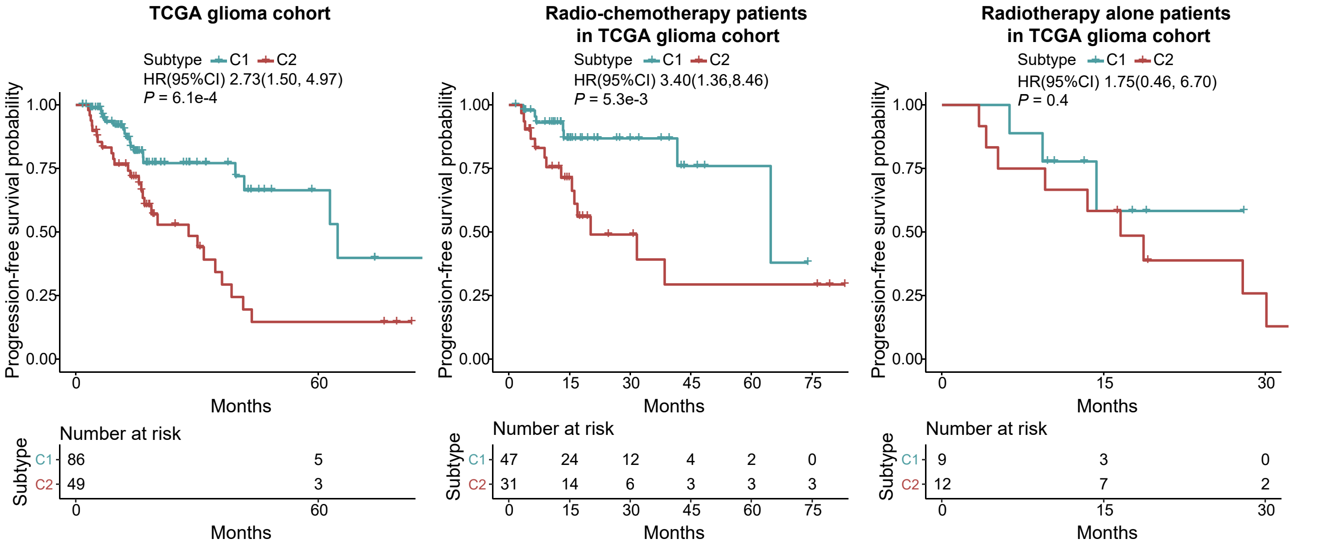

TCGA glioma cohort
Subtype C1 C2
HR(95%CI) 2.73(1.50, 4.97)
P = 6.1e-4
Progression-free survival probability
1.00
0.75
0.50
0.25
0.00
0
60
Months
Number at risk
Subtype
C1 86 5
C2 49 3
0
60
Months
Radio-chemotherapy patients in TCGA glioma cohort
Subtype C1 C2
HR(95%CI) 3.40(1.36,8.46)
P = 5.3e-3
Progression-free survival probability
1.00
0.75
0.50
0.25
0.00
0 15 30 45 60 75
Months
Number at risk
Subtype
C1 47 24 12 4 2 0
C2 31 14 6 3 3 3
0 15 30 45 60 75
Months
Radiotherapy alone patients in TCGA glioma cohort
Subtype C1 C2
HR(95%CI) 1.75(0.46, 6.70)
P = 0.4
Progression-free survival probability
1.00
0.75
0.50
0.25
0.00
0 15 30
Months
Number at risk
Subtype
C1 9 3 0
C2 12 7 2
0 15 30
Months
