# Supplementary material for: Transcriptomic Analysis Identified Two Subtypes of Brain Tumor Characterized by Distinct Immune Infiltration and Prognosis
Source: Front Oncol. 2021 Oct 15;11:734407. doi: 10.3389/fonc.2021.734407 (PMC8554158; doi:10.3389/fonc.2021.734407)

Combined cohort

Variable	*N*	Hazard ratio	*P*
**Age**	696	1.01 (1.00, 1.02)	0.005
**Gender**			
Female	279	Reference	
Male	417	0.91 (0.75, 1.10)	0.333
**Grade**			
II	183	Reference	
III	218	1.84 (1.34, 2.53)	<0.001
IV	295	4.25 (2.50, 7.22)	<0.001
**Histology**			
Glioblastoma	295	Reference	
Astrocytoma	263	1.56 (0.96, 2.54)	0.070
Oligoastrocytoma	0		
Oligodendroglioma	138		
***IDH* mutation**			
Yes	375	Reference	
No	321	1.45 (1.13, 1.86)	0.003
**Co-deletion of 1p and 19q**			
No	552	Reference	
Yes	144	0.46 (0.29, 0.72)	<0.001
***MGMT* methylation**			
No	311	Reference	
Yes	385	0.78 (0.64, 0.94)	0.010
**Therapy**			
Radio−chemotherapy	465	Reference	
Chemotherapy	98	0.70 (0.51, 0.97)	0.032
Radiotherapy	133	1.41 (1.08, 1.83)	0.011
**Cancer type**			
Primary	460	Reference	
Recurrent	208	2.51 (2.02, 3.12)	<0.001
Secondary	28	4.13 (2.64, 6.48)	<0.001
**Subtype**			
C1	256	Reference	
C2	440	2.24 (1.74, 2.88)	<0.001

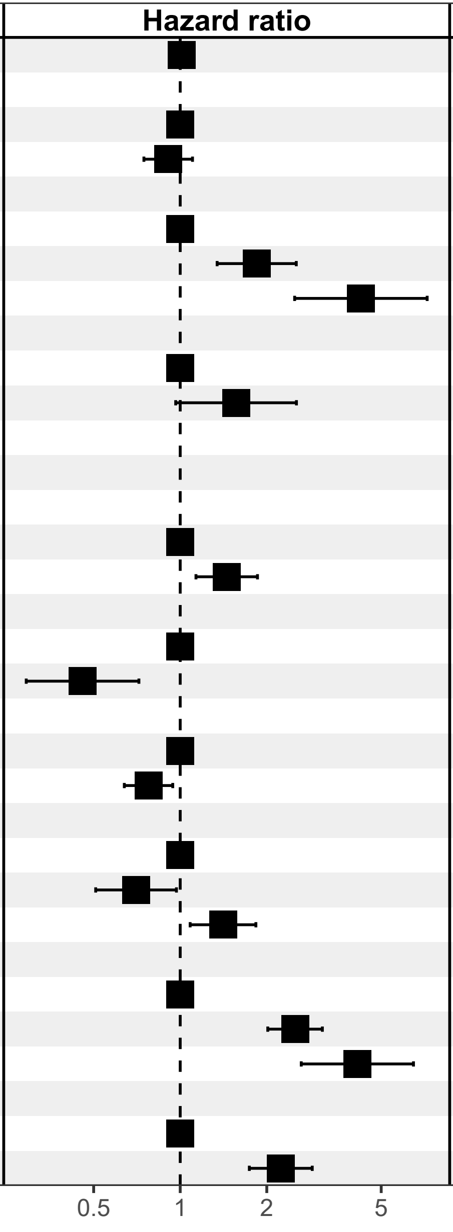

CGGA-C cohort

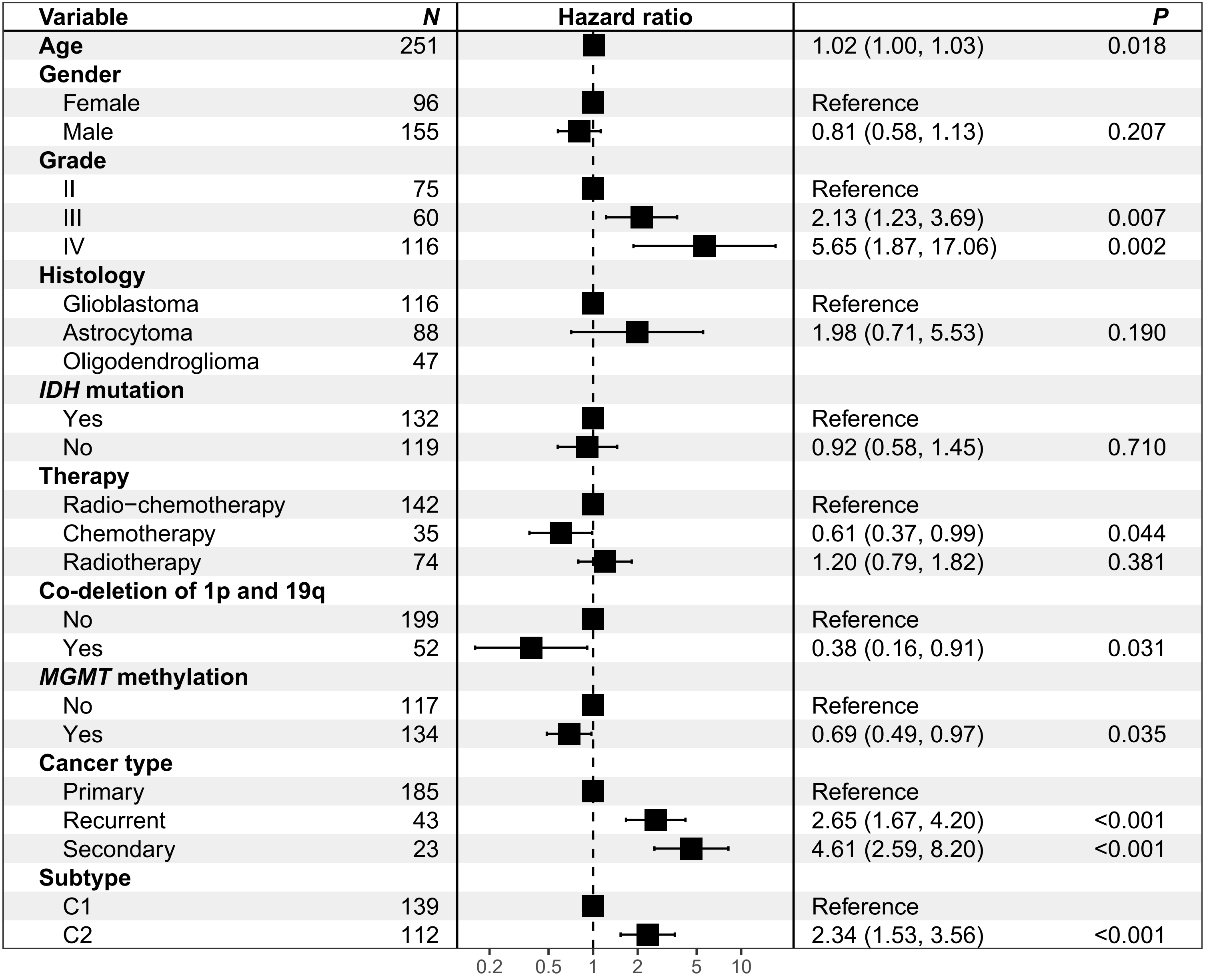

CGGA-B cohort

Variable	*N*		*P*
**Age**	368	1.01 (1.00, 1.02)	0.04
**Gender**			
Female	155	Reference	
Male	213	1.14 (0.87, 1.49)	0.33
**Grade**			
II	82	Reference	
III	141	2.24 (1.41, 3.56)	<0.001
IV	145	2.81 (1.65, 4.79)	<0.001
**Histology**			
Astrocytoma	150	Reference	
Glioblastoma	145		
Oligoastrocytoma	0		
Oligodendroglioma	73	0.63 (0.35, 1.14)	0.12
***IDH* mutation**			
Yes	202	Reference	
No	166	1.79 (1.27, 2.52)	<0.001
**Therapy**			
Radio−chemotherapy	284	Reference	
Chemotherapy	40	1.10 (0.69, 1.75)	0.70
Radiotherapy	44	1.51 (0.99, 2.31)	0.05
**Co-deletion of 1p and 19q**			
No	292	Reference	
Yes	76	0.65 (0.36, 1.15)	0.14
***MGMT* methylation**			
No	154	Reference	
Yes	214	0.83 (0.64, 1.09)	0.18
**Cancer type**			
Primary	217	Reference	
Recurrent	151	2.31 (1.76, 3.03)	<0.001
**Subtype**			
C1	199	Reference	
C2	169	1.78 (1.28, 2.47)	<0.001

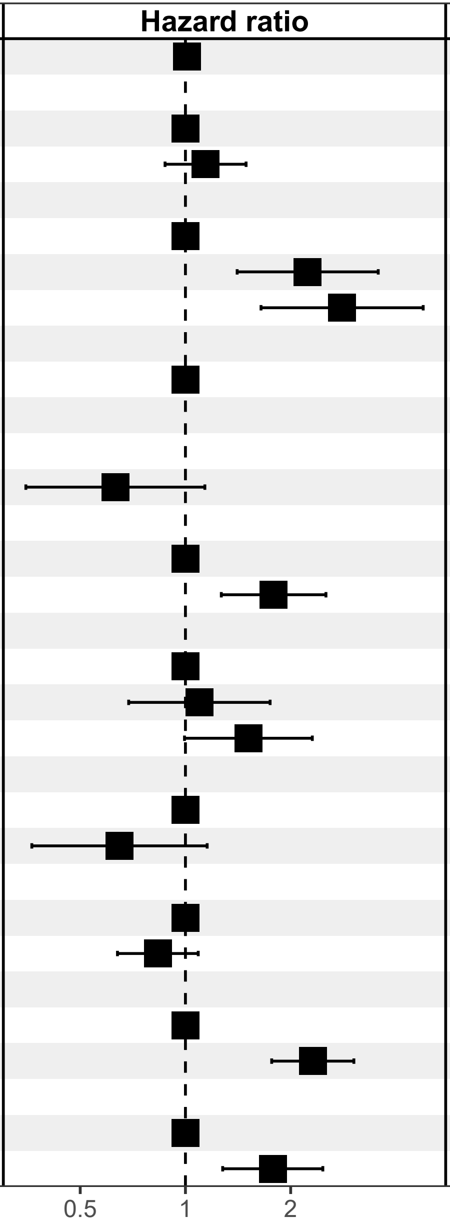

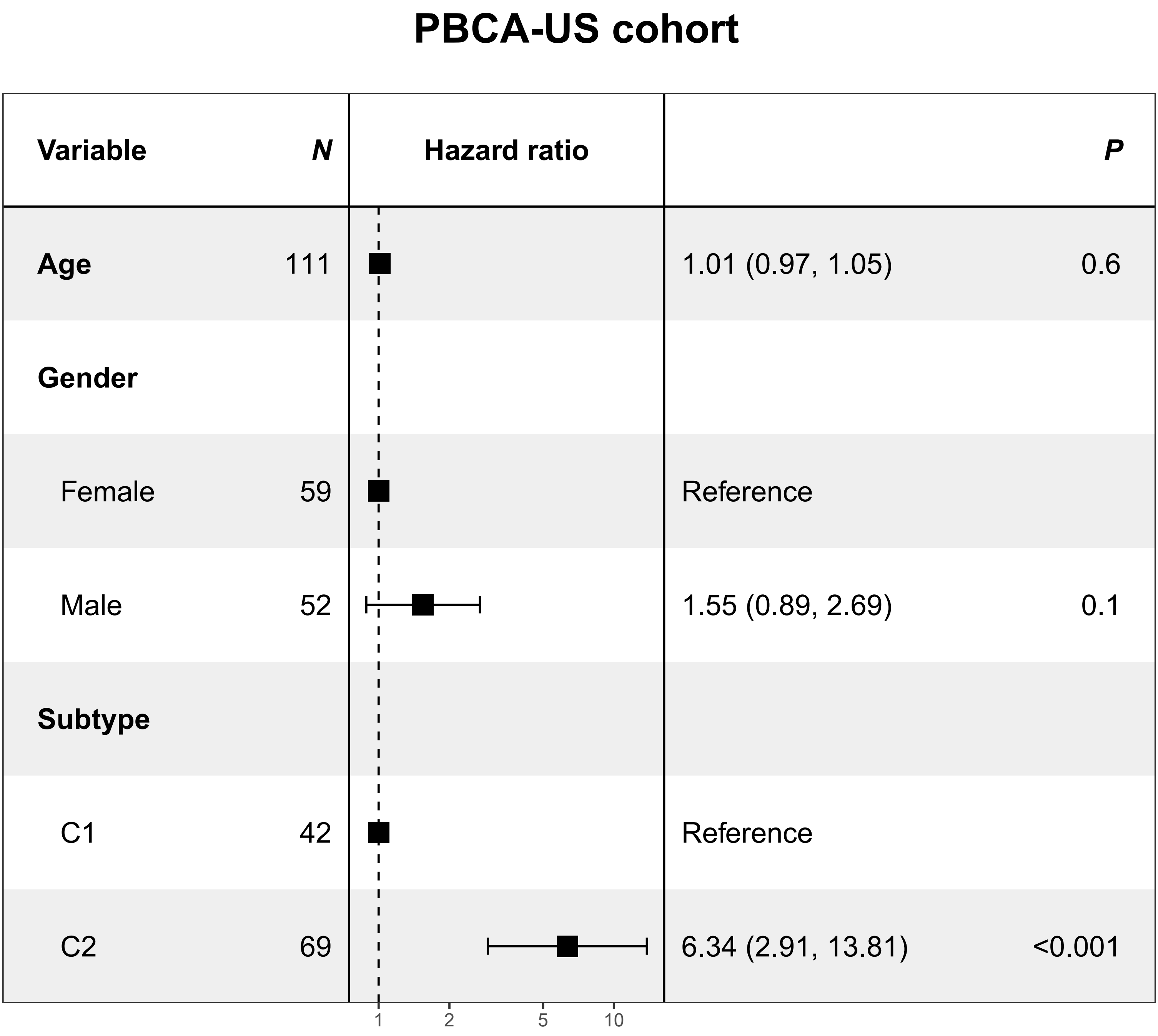
PBCA-US cohort
Variable
N
Hazard ratio
P
Age
111
1.01 (0.97, 1.05)
0.6
Gender
Female
59
Reference
Male
52
1.55 (0.89, 2.69)
0.1
Subtype
C1
42
Reference
C2
69
6.34 (2.91, 13.81)
<0.001
1
2
5
10

CGGA-D cohort

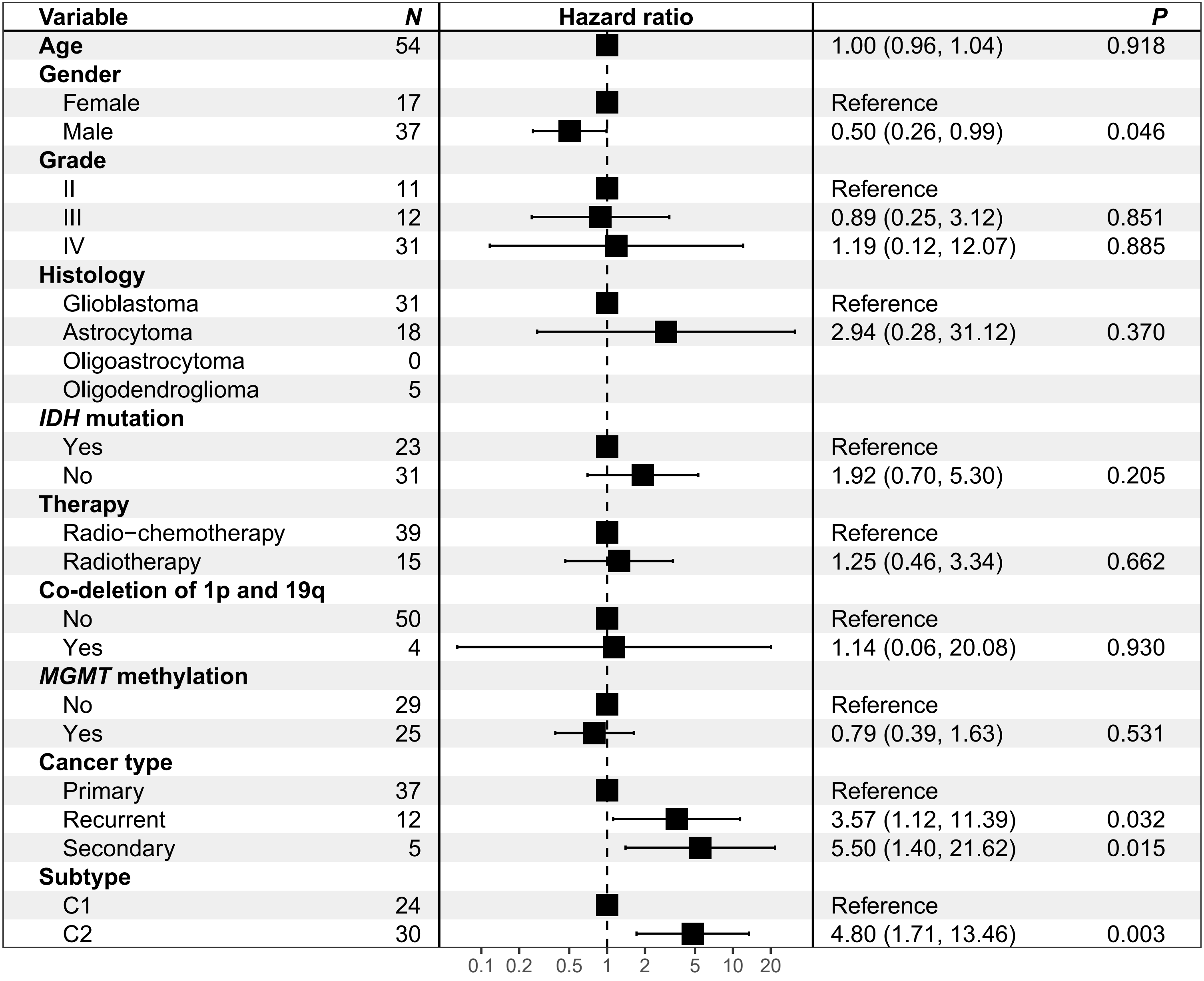

GSE16011 cohort

Variable	*N*	Hazard ratio	*P*
**Age**	105	1.04 (1.02, 1.06)	<0.001
**Gender**			
Female	34	Reference	
Male	71	1.07 (0.67, 1.72)	0.77
**Grade**			
II	13	Reference	
III	46	0.74 (0.32, 1.73)	0.49
IV	46	1.08 (0.43, 2.76)	0.87
**Histology**			
Astrocytoma	11	Reference	
Glioblastoma	46		
Oligoastrocytoma	9	0.90 (0.30, 2.75)	0.86
Oligodendroglioma	39	0.71 (0.26, 1.90)	0.49
***IDH* mutation**			
Yes	44	Reference	
No	61	1.58 (0.94, 2.65)	0.08
**Co-deletion of 1p and 19q**			
No	68	Reference	
Yes	37	0.72 (0.39, 1.31)	0.28
**Subtype**			
C1	44	Reference	
C2	61	1.55 (0.91, 2.67)	0.11

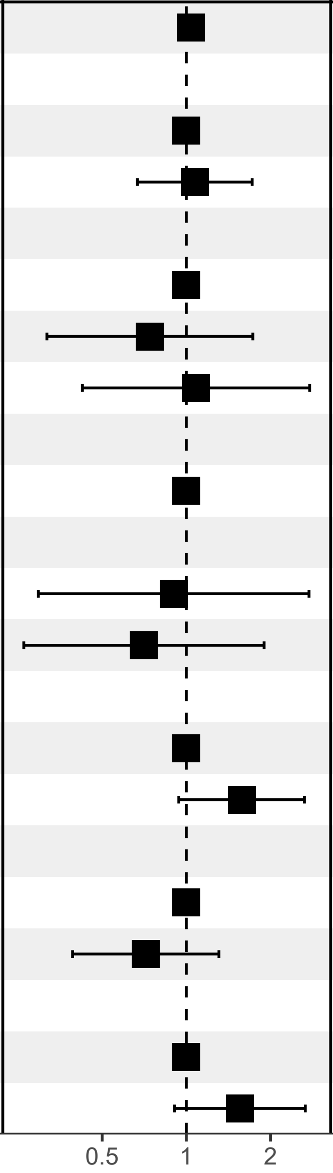

TCGA-LGG cohort

Variable	*N*	Hazard ratio	*P*
**Age**	272	1.06 (1.04, 1.09)	<0.001
**Gender**			
Female	124	Reference	
Male	148	1.14 (0.68, 1.91)	0.621
**Grade**			
II	132	Reference	
III	140	2.49 (1.32, 4.70)	0.005
**Histology**			
Astrocytoma	94	Reference	
Oligoastrocytoma	68	1.67 (0.87, 3.22)	0.125
Oligodendroglioma	110	0.89 (0.45, 1.75)	0.741
***IDH* mutation**			
Yes	215	Reference	
No	57	2.54 (1.07, 6.04)	0.034
**Co-deletion of 1p and 19q**			
Yes	81	Reference	
No	191	1.54 (0.69, 3.43)	0.288
***MGMT* methylation**			
Yes	218	Reference	
No	54	0.84 (0.39, 1.81)	0.654
**Surgery**			
Gross Total Resection	171	Reference	
Subtotal Resection	101	1.21 (0.70, 2.09)	0.500
**Subtype**			
C1	172	Reference	
C2	100	1.78 (1.00, 3.16)	0.051

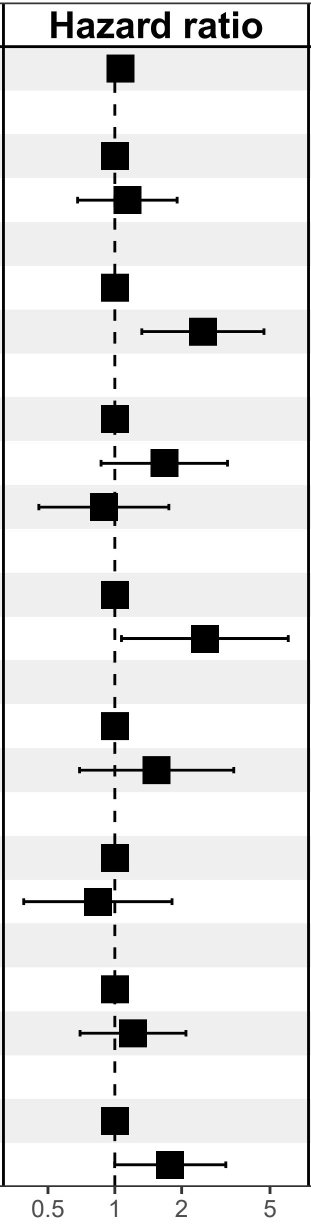

GSE108474 cohort

Variable	*N*	Hazard ratio	*P*
**Age**			
15−49	123	Reference	
50−89	108	2.07 (1.50, 2.86)	<0.001
**Gender**			
Female	89	Reference	
Male	142	1.16 (0.85, 1.58)	0.353
**Grade**			
II	68	Reference	
III	63	1.37 (0.93, 2.02)	0.110
IV	100	2.07 (1.17, 3.66)	0.012
**Histology**			
Glioblastoma	100	Reference	
Astrocytoma	88	0.99 (0.62, 1.60)	0.975
Oligoastrocytoma	6	0.77 (0.29, 2.03)	0.592
Oligodendroglioma	37		
**Subtype**			
C1	97	Reference	
C2	134	1.56 (1.12, 2.18)	0.009

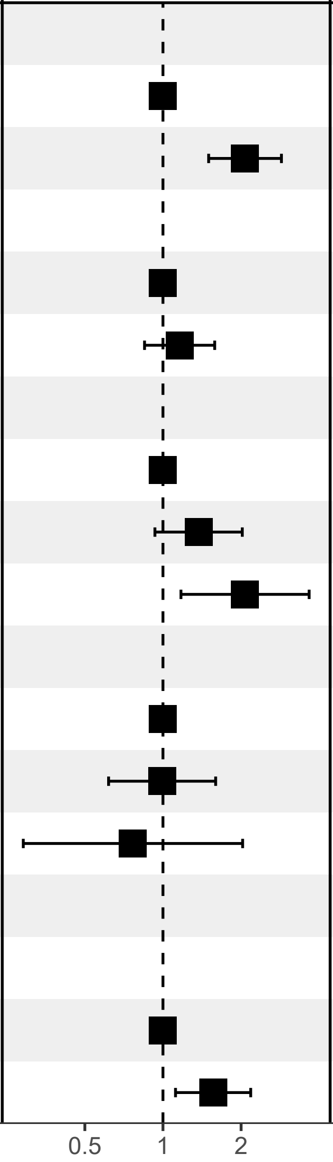

E-MTAB-3892 cohort

Variable	*N*	Hazard ratio	*P*
**Age**	150	1.04 (1.01, 1.07)	0.012
**Gender**			
Female	63	Reference	
Male	87	0.89 (0.46, 1.72)	0.727
**Grade**			
II	43	Reference	
III	89	1.16 (0.49, 2.74)	0.731
IV	18	10.31 (1.28, 83.01)	0.028
**Histology**			
Glioblastoma	14	Reference	
Astrocytoma	2	2.53 (0.12, 54.04)	0.553
Oligoastrocytoma	33	2.09 (0.40, 10.78)	0.380
Oligodendroglioma	101	7.96 (0.92, 69.15)	0.060
***IDH* mutation**			
Yes	116	Reference	
No	34	2.15 (0.80, 5.77)	0.130
**Co-deletion of 1p and 19q**			
Yes	87	Reference	
No	63	4.15 (1.56, 11.09)	0.004
**Subtype**			
C1	117	Reference	
C2	33	0.96 (0.37, 2.52)	0.941

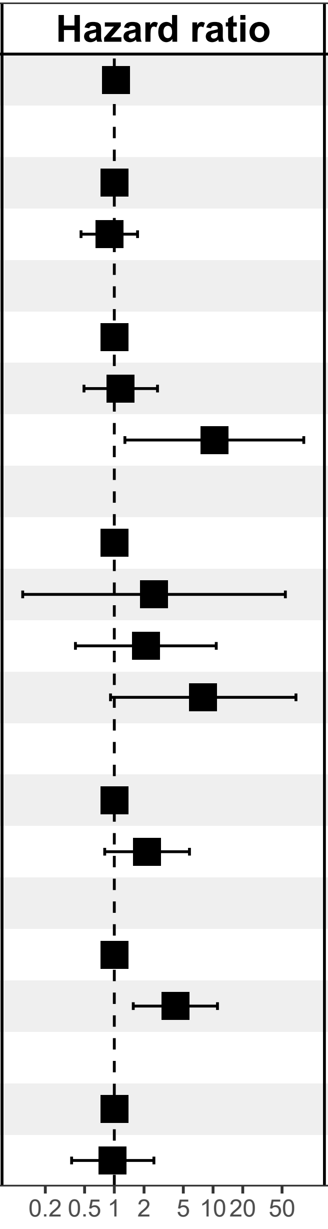

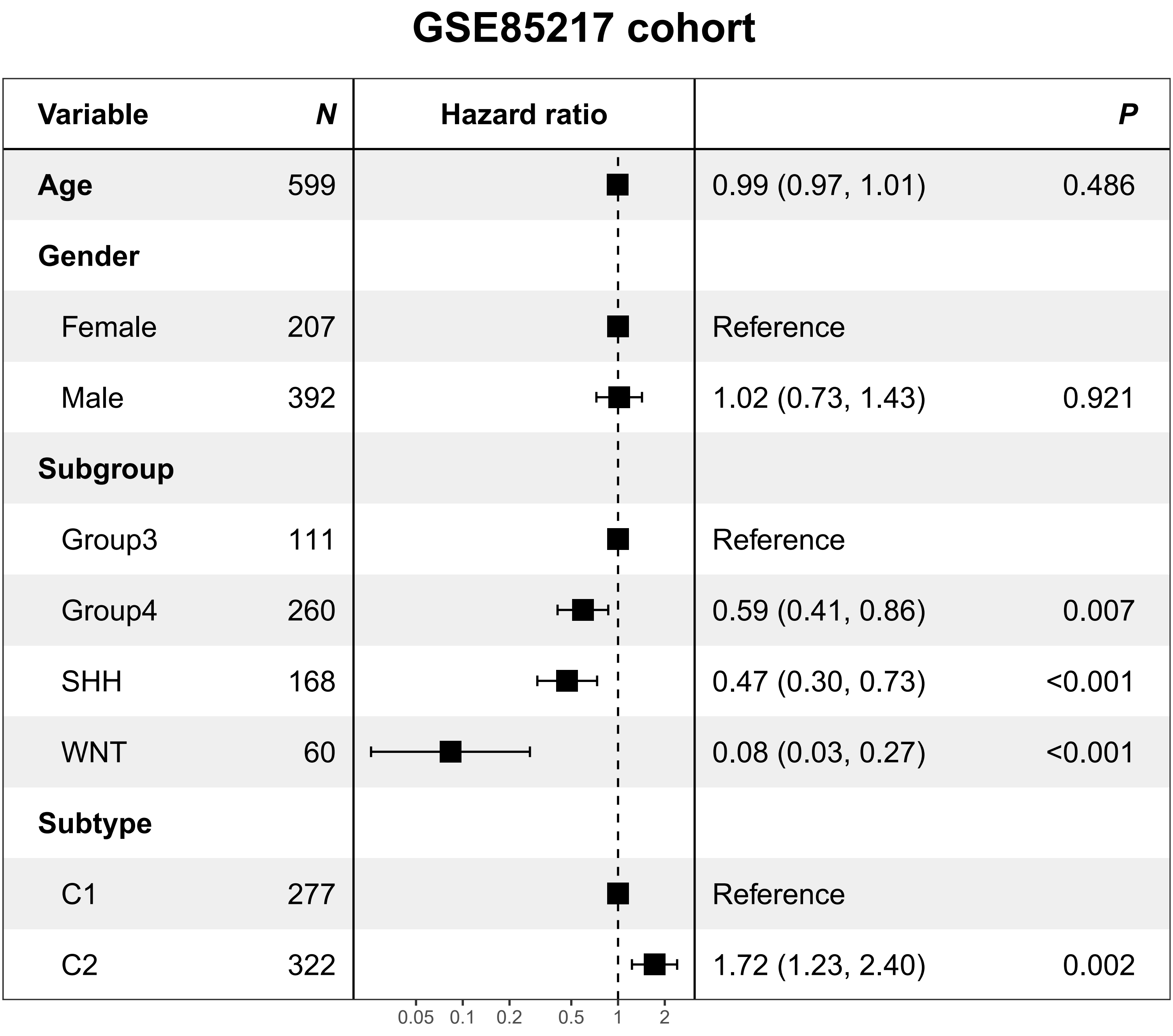
GSE85217 cohort
Variable
N
Hazard ratio
P
Age
599
0.99 (0.97, 1.01)
0.486
Gender
Female
207
Reference
Male
392
1.02 (0.73, 1.43)
0.921
Subgroup
Group3
111
Reference
Group4
260
0.59 (0.41, 0.86)
0.007
SHH
168
0.47 (0.30, 0.73)
<0.001
WNT
60
0.08 (0.03, 0.27)
<0.001
Subtype
C1
277
Reference
C2
322
1.72 (1.23, 2.40)
0.002
0.05
0.1
0.2
0.5
1
2

TCGA-GBM Cohort

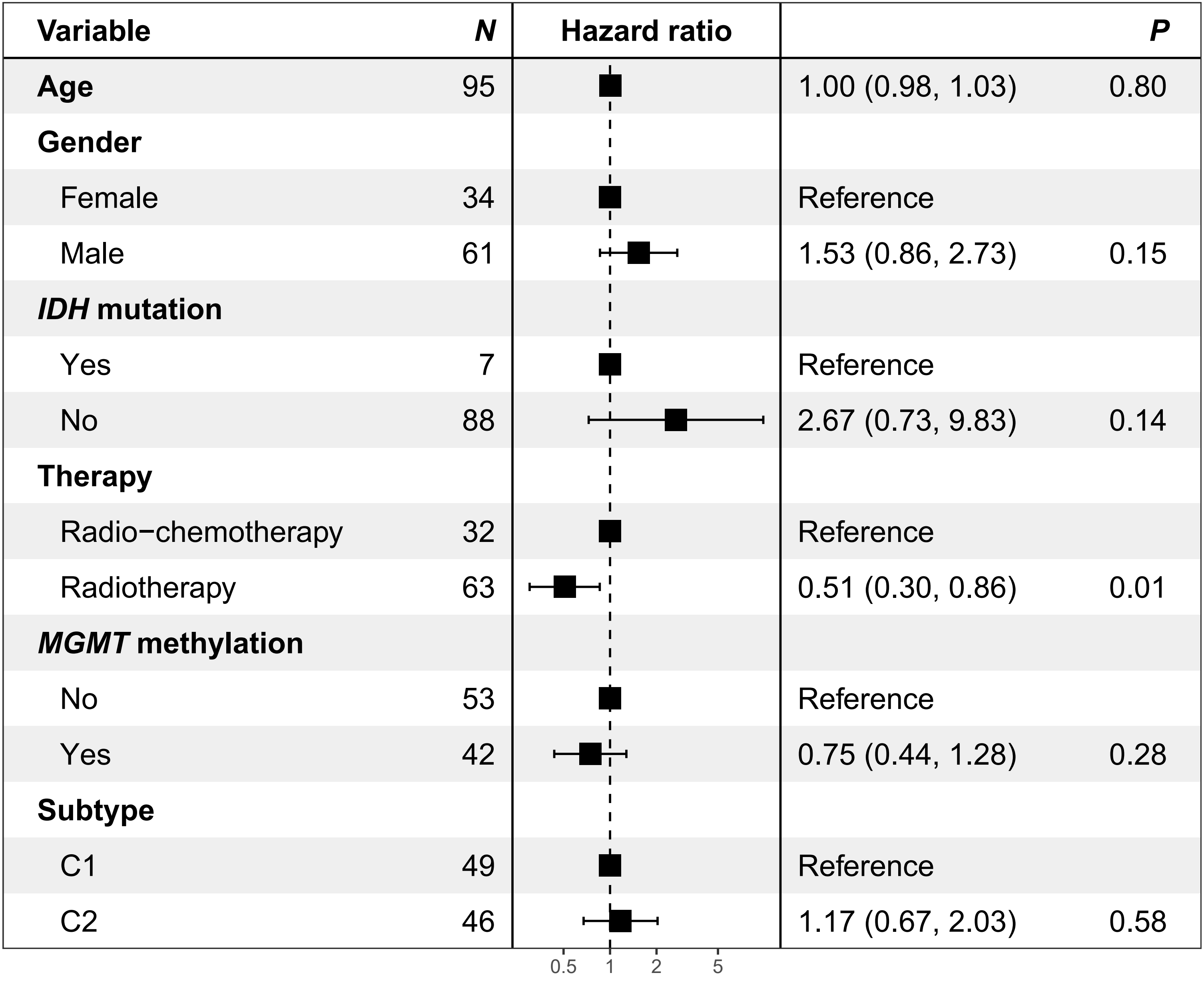

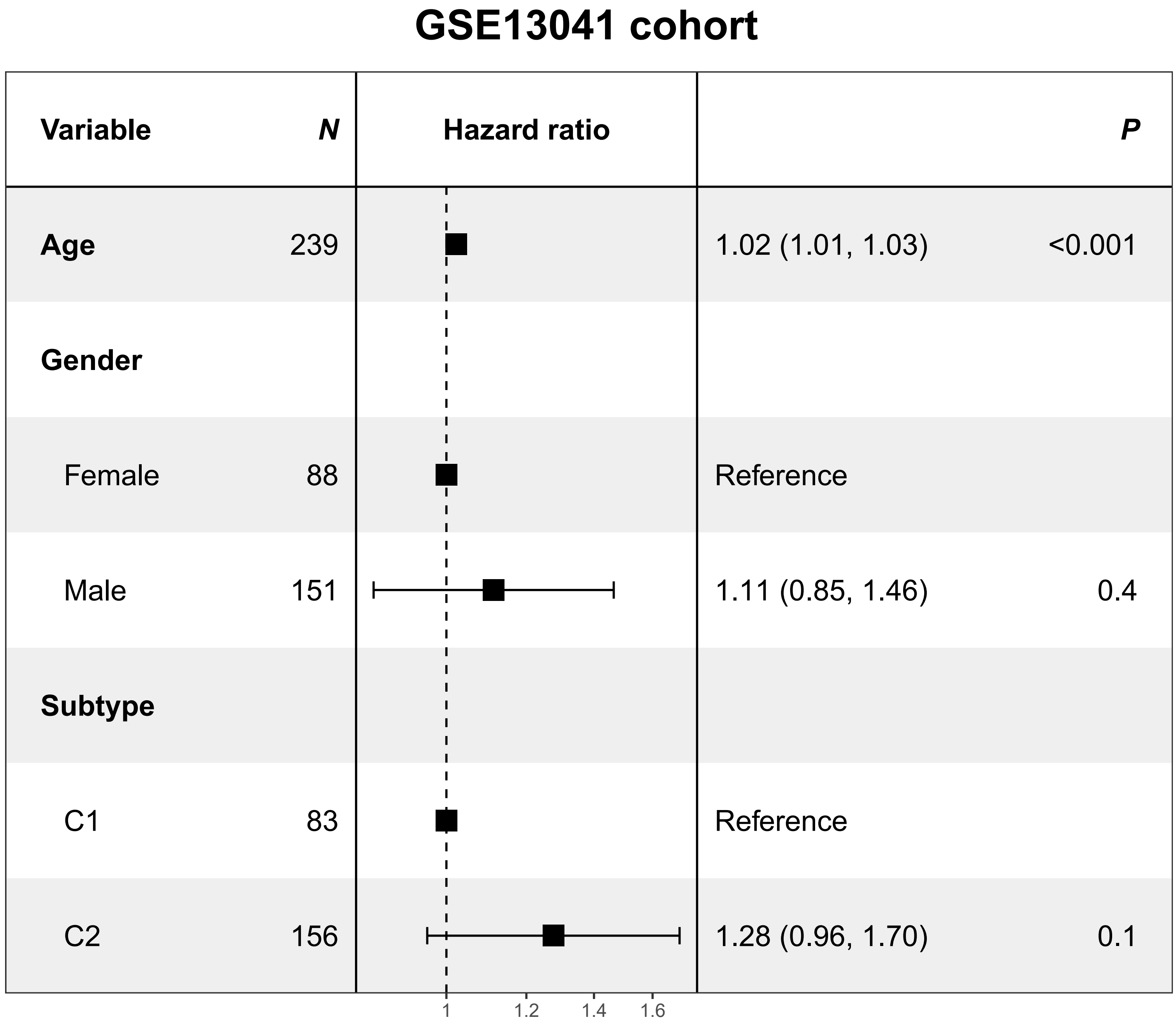
GSE13041 cohort
Variable
N
Hazard ratio
P
Age
239
1.02 (1.01, 1.03)
<0.001
Gender
Female
88
Reference
Male
151
1.11 (0.85, 1.46)
0.4
Subtype
C1
83
Reference
C2
156
1.28 (0.96, 1.70)
0.1
1
1.2
1.4
1.6

Astrocytoma subgroup

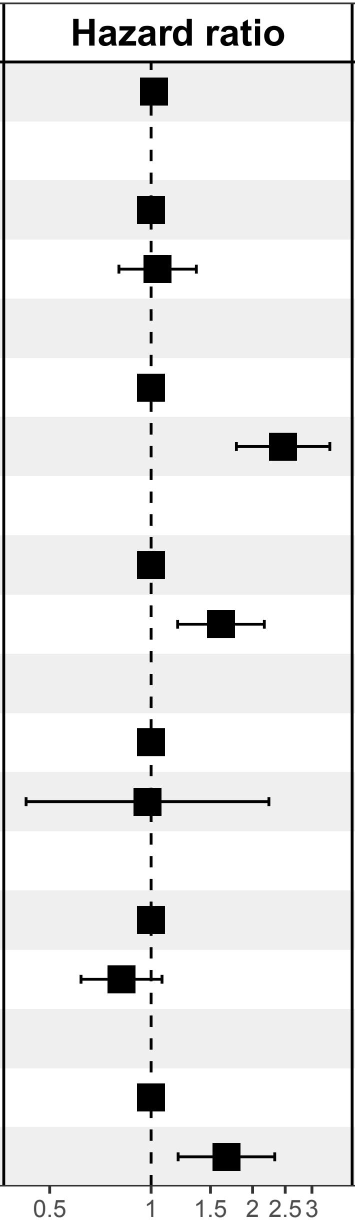

Variable	N	Hazard ratio	*P*
**Age**	473	1.02 (1.01, 1.03)	0.002
**Gender**			
Female	188	Reference	
Male	285	1.05 (0.80, 1.36)	0.742
**Grade**			
II	180	Reference	
III	293	2.46 (1.79, 3.39)	<0.001
***IDH* mutation**			
Yes	319	Reference	
No	154	1.61 (1.20, 2.17)	0.002
**Co-deletion of 1p and 19q**			
No	460	Reference	
Yes	13	0.98 (0.43, 2.24)	0.953
***MGMT* methylation**			
No	191	Reference	
Yes	282	0.82 (0.62, 1.08)	0.151
**Subtype**			
C1	157	Reference	
C2	316	1.67 (1.20, 2.33)	0.002

Oligodendroglioma subgroup

Variable	N	Hazard ratio		*P*
**Age**	330		1.05 (1.03, 1.08)	<0.001
**Gender**				
Female	158		Reference	
Male	172		1.22 (0.77, 1.93)	0.406
**Grade**				
II	185		Reference	
III	145		2.06 (1.25, 3.41)	0.005
***IDH* mutation**				
Yes	298		Reference	
No	32		1.53 (0.63, 3.70)	0.346
**Co-deletion of 1p and 19q**				
No	80		Reference	
Yes	250		0.43 (0.22, 0.85)	0.016
***MGMT* methylation**				
No	72		Reference	
Yes	258		0.90 (0.50, 1.62)	0.717
**Subtype**				
C1	102		Reference	
C2	228		2.60 (1.25, 5.40)	0.010

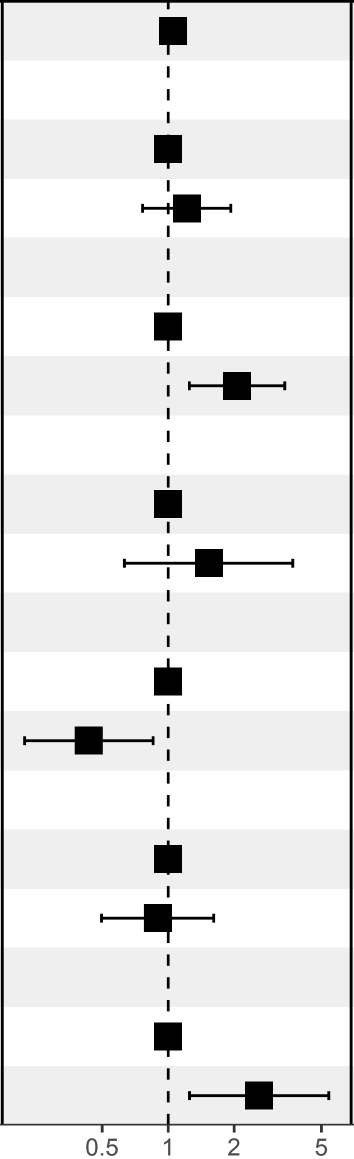

IDH wildtype subgroup

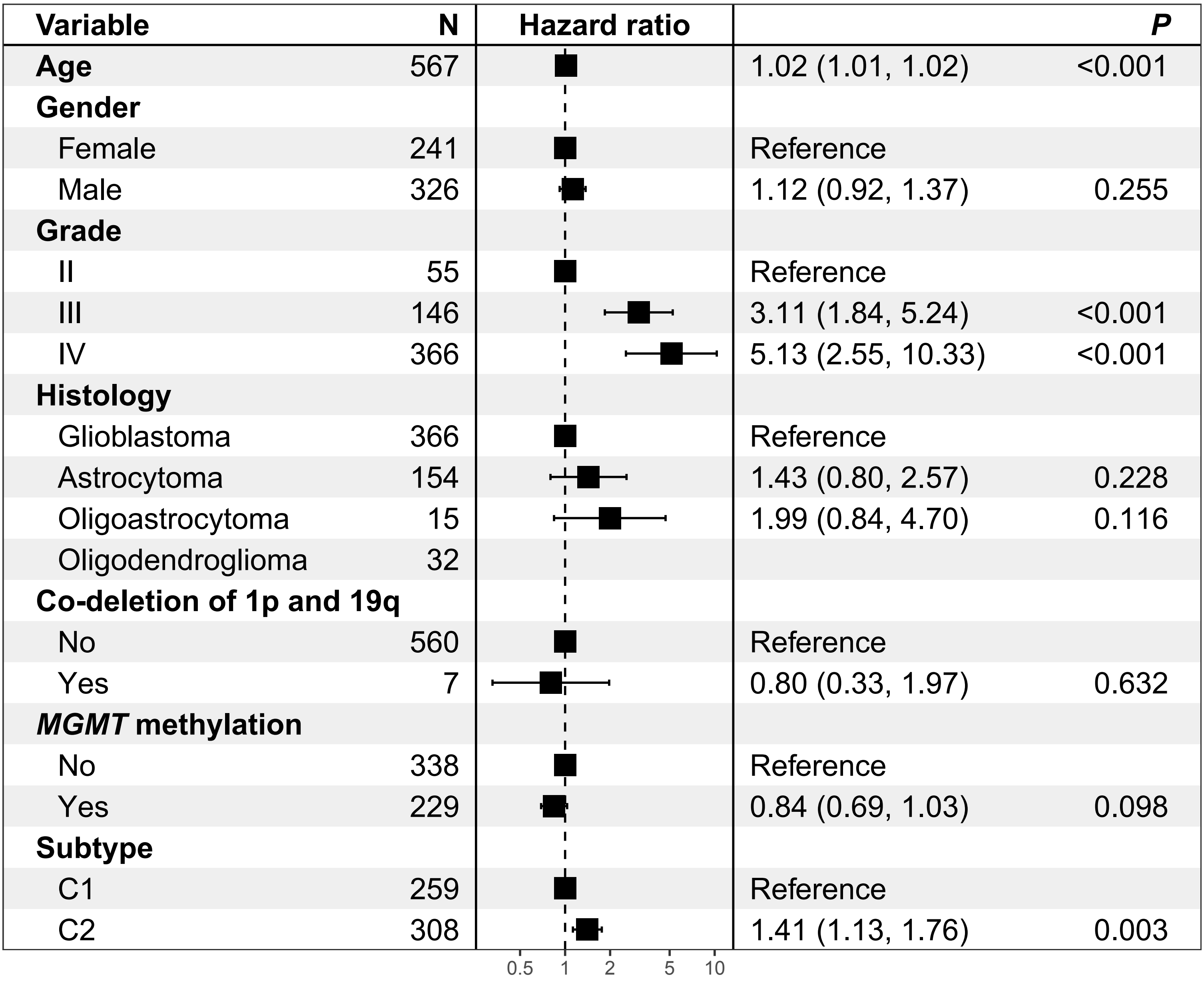

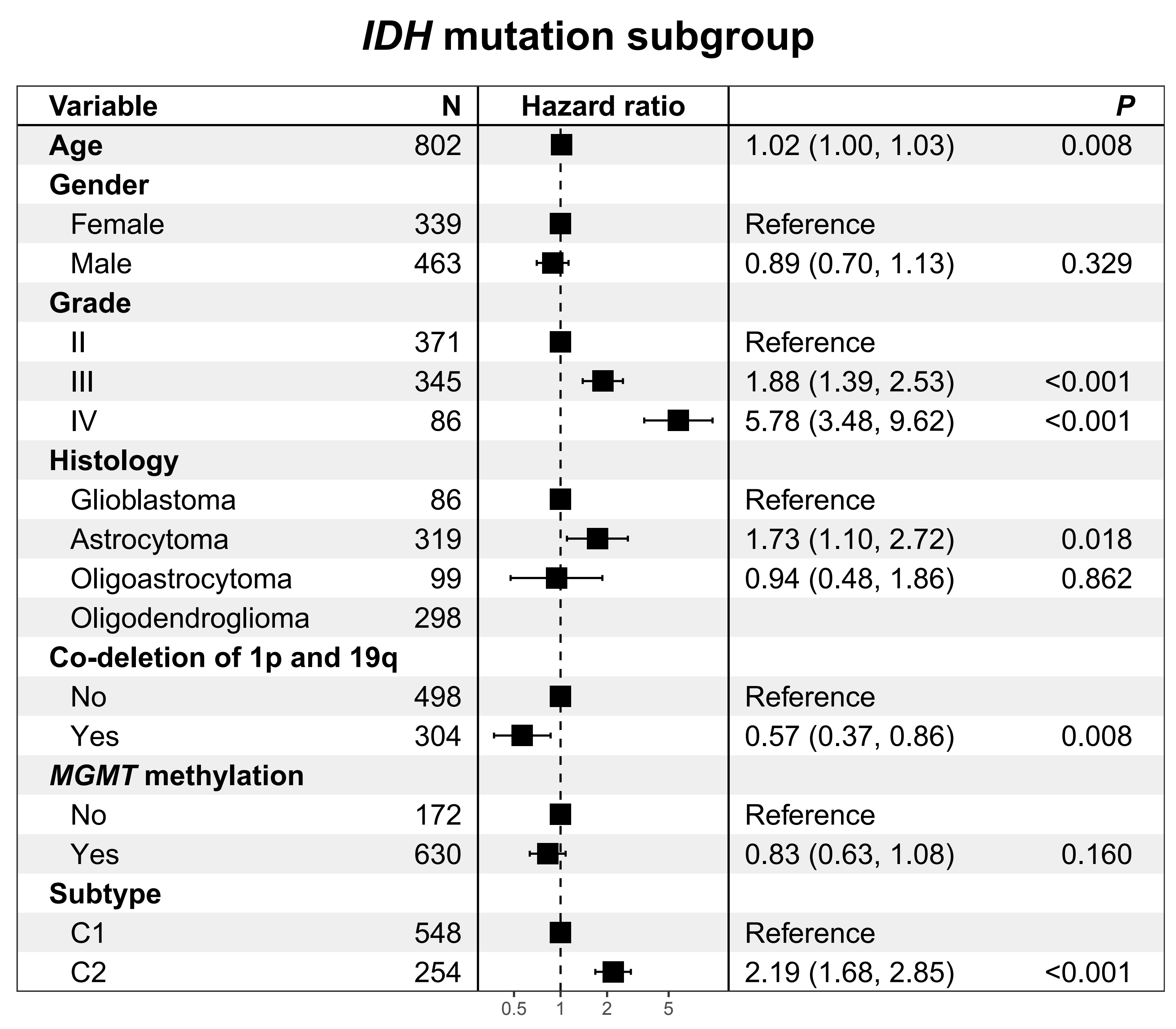
IDH mutation subgroup
Variable
N
Hazard ratio
P
Age
802
1.02 (1.00, 1.03)
0.008
Gender
Female
339
Reference
Male
463
0.89 (0.70, 1.13)
0.329
Grade
II
371
Reference
III
345
1.88 (1.39, 2.53)
<0.001
IV
86
5.78 (3.48, 9.62)
<0.001
Histology
Glioblastoma
86
Reference
Astrocytoma
319
1.73 (1.10, 2.72)
0.018
Oligoastrocytoma
99
0.94 (0.48, 1.86)
0.862
Oligodendroglioma
298
Co-deletion of 1p and 19q
No
498
Reference
Yes
304
0.57 (0.37, 0.86)
0.008
MGMT methylation
No
172
Reference
Yes
630
0.83 (0.63, 1.08)
0.160
Subtype
C1
548
Reference
C2
254
2.19 (1.68, 2.85)
<0.001
0.5
1
2
5

IDH mutation with 1p/19q- subgroup

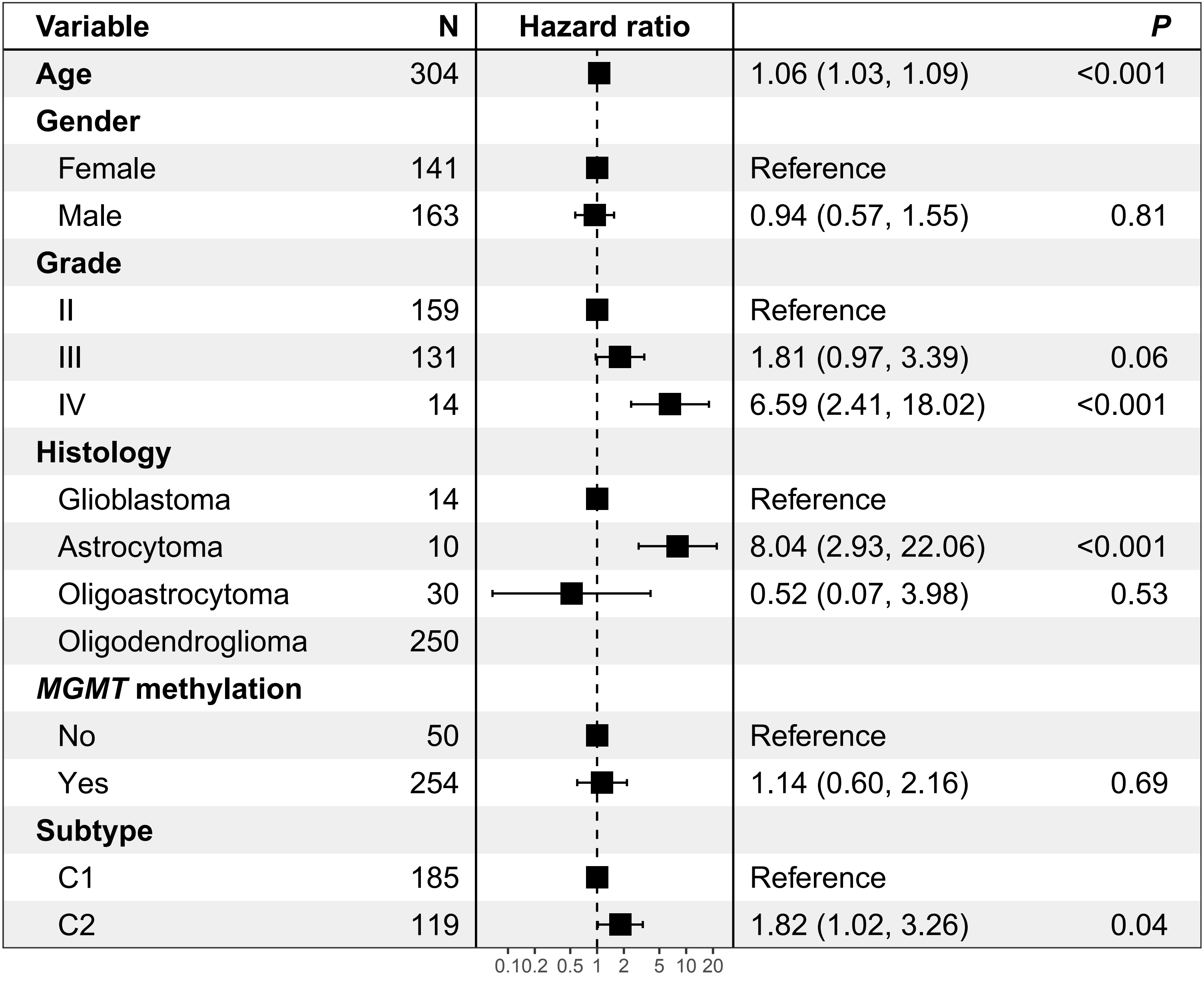

IDH mutation without 1p/19q- subgroup

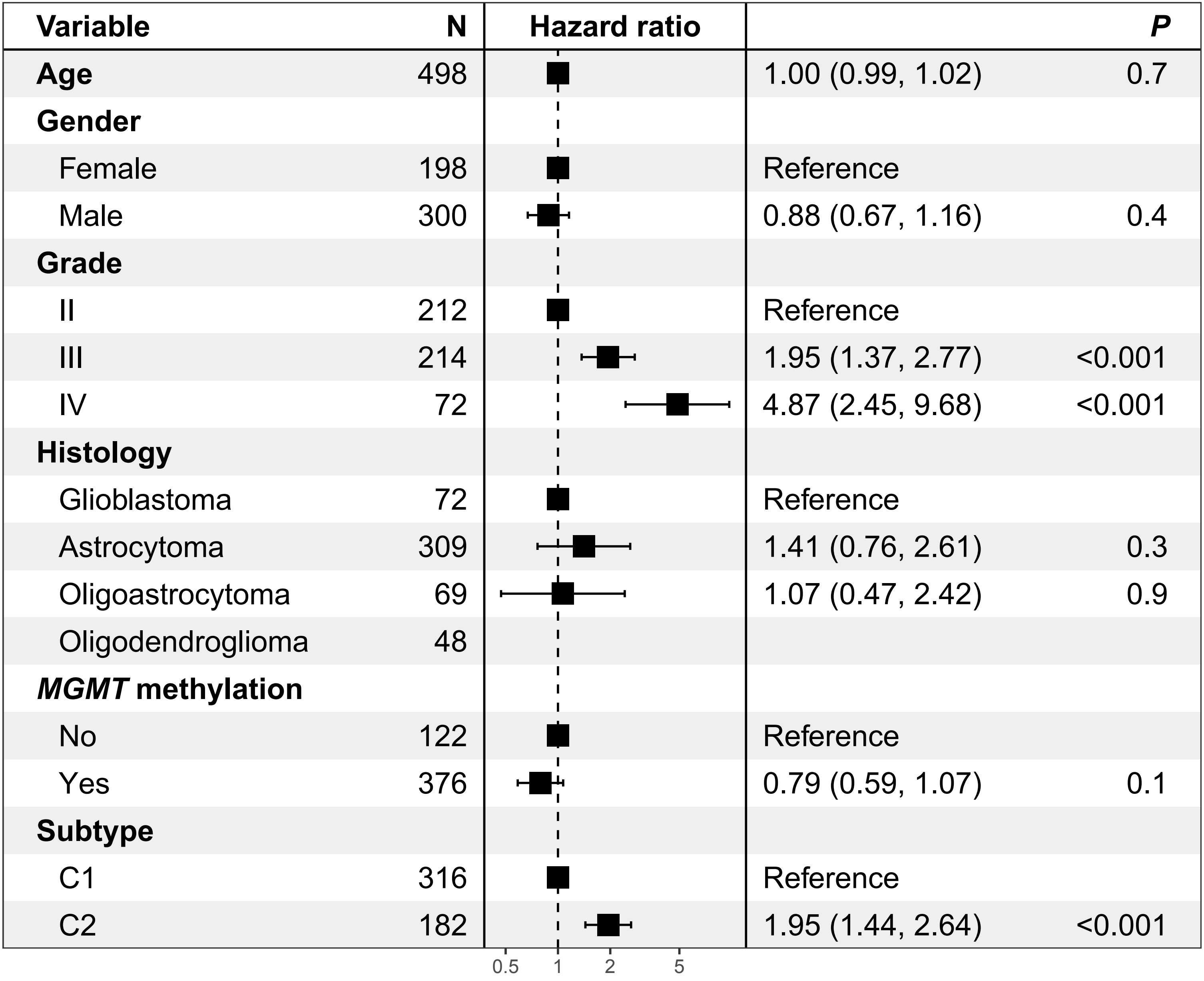

Tumor grade II subgroup

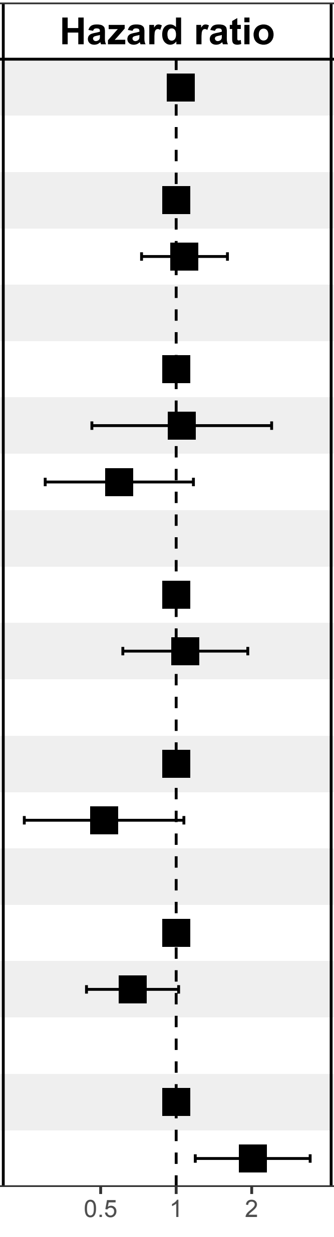

Variable	N	Hazard ratio	*P*
**Age**	426	1.04 (1.02, 1.06)	<0.001
**Gender**			
Female	190	Reference	
Male	236	1.08 (0.73, 1.60)	0.704
**Histology**			
Astrocytoma	180	Reference	
Oligoastrocytoma	61	1.05 (0.46, 2.40)	0.903
Oligodendroglioma	185	0.59 (0.30, 1.17)	0.133
***IDH* mutation**			
Yes	371	Reference	
No	55	1.09 (0.61, 1.93)	0.774
**Co-deletion of 1p and 19q**			
No	267	Reference	
Yes	159	0.52 (0.25, 1.07)	0.077
***MGMT* methylation**			
No	138	Reference	
Yes	288	0.67 (0.44, 1.02)	0.063
**Subtype**			
C1	129	Reference	
C2	297	2.02 (1.19, 3.42)	0.009

Tumor grade III subgroup

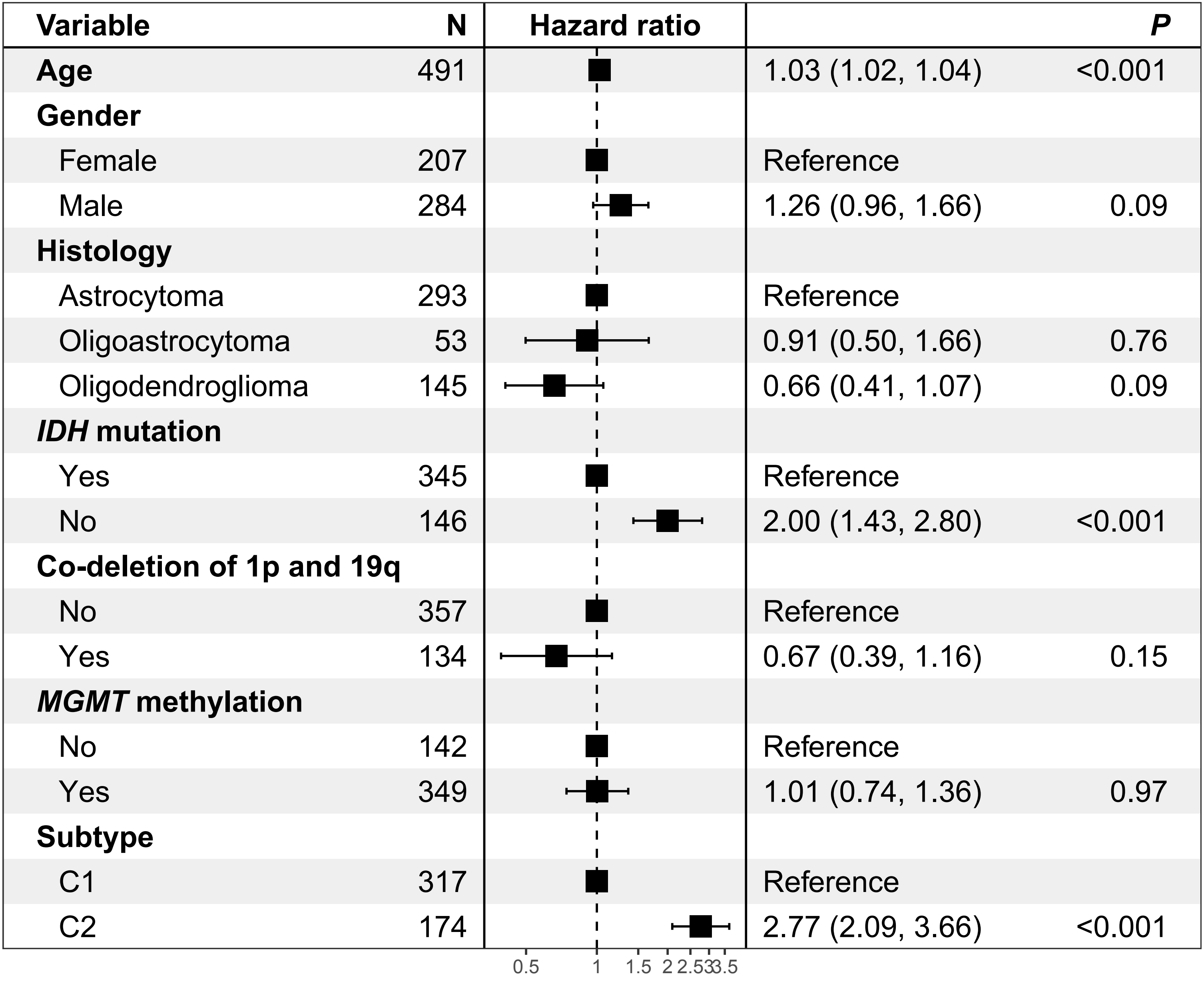

Tumor grade IV cohort

Variable	N	Hazard ratio	*P*
**Age**	452	1.01 (1.00, 1.02)	0.007
**Gender**			
Female	183	Reference	
Male	269	0.97 (0.78, 1.20)	0.783
***IDH* mutation**			
Yes	86	Reference	
No	366	1.12 (0.82, 1.52)	0.471
**Co-deletion of 1p and 19q**			
No	434	Reference	
Yes	18	0.64 (0.35, 1.15)	0.134
***MGMT* methylation**			
No	230	Reference	
Yes	222	0.82 (0.66, 1.02)	0.072
**Subtype**			
C1	272	Reference	
C2	180	1.25 (0.98, 1.59)	0.073

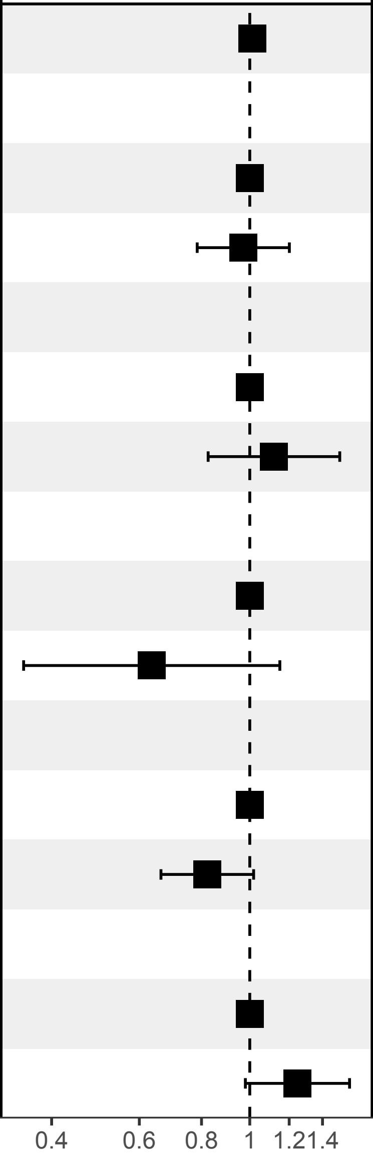

Supplement: Supplementary Figure 1 — A perspective of contrastive learning. V0, V0’ are two different views of the same sample X0. The feature encoder represents V0 and V0’ in a reduced dimensional space as R0 and R0’ . Contrastive learning algorithm trains the feature encoder by driving the maximum similarity between R0 and R0’ . [file DataSheet_1.zip › Supplementary material/Figure S12.pdf]
