# Supplementary figures and images for: Transcriptomic Analysis Identified Two Subtypes of Brain Tumor Characterized by Distinct Immune Infiltration and Prognosis
Source: Front Oncol. 2021 Oct 15;11:734407. doi: 10.3389/fonc.2021.734407 (PMC8554158; doi:10.3389/fonc.2021.734407)

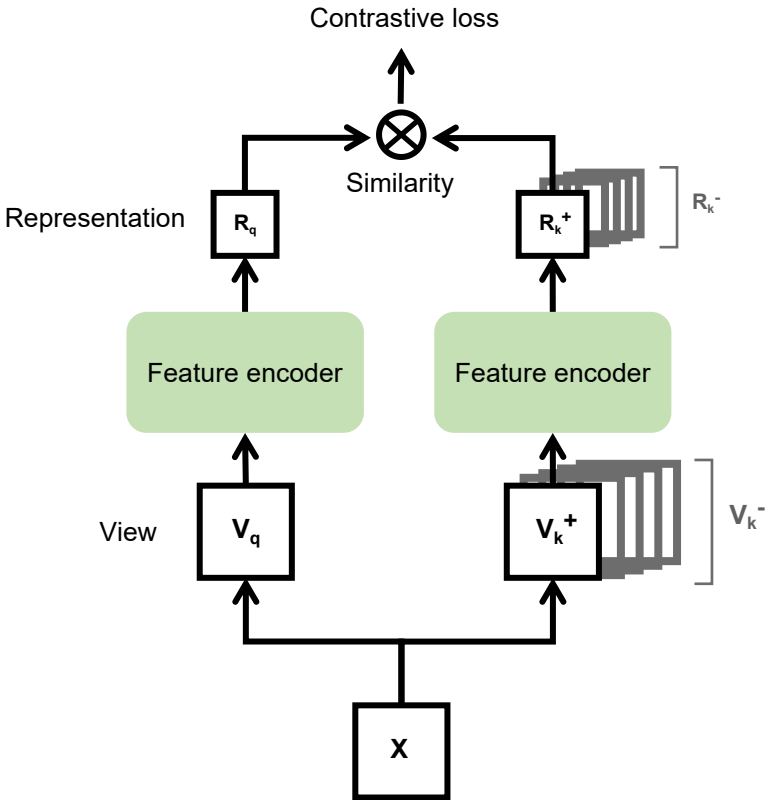

Supplement: Supplementary Figure 1 — A perspective of contrastive learning. V0, V0’ are two different views of the same sample X0. The feature encoder represents V0 and V0’ in a reduced dimensional space as R0 and R0’ . Contrastive learning algorithm trains the feature encoder by driving the maximum similarity between R0 and R0’ . [file DataSheet_1.zip › Supplementary material/Figure S1.pdf]

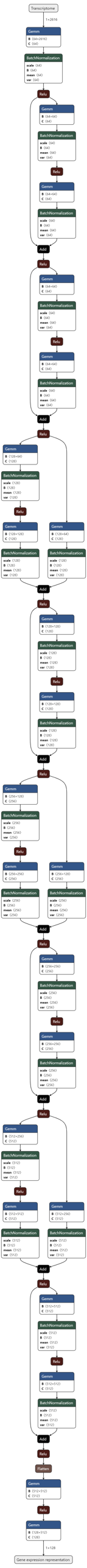

Supplement: Supplementary Figure 1 — A perspective of contrastive learning. V0, V0’ are two different views of the same sample X0. The feature encoder represents V0 and V0’ in a reduced dimensional space as R0 and R0’ . Contrastive learning algorithm trains the feature encoder by driving the maximum similarity between R0 and R0’ . [file DataSheet_1.zip › Supplementary material/Figure S2.pdf]

A

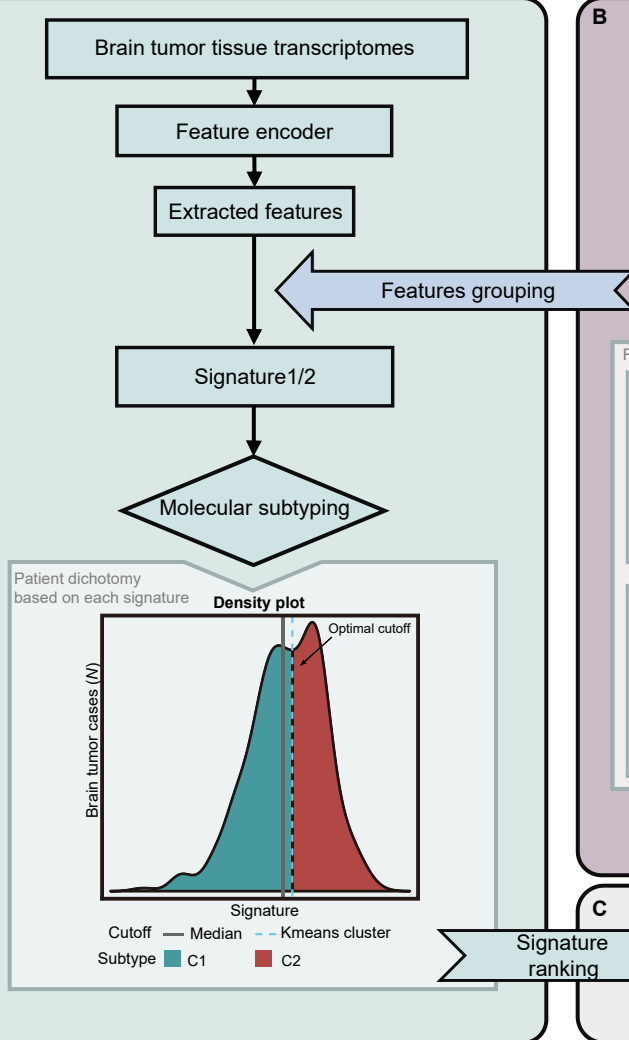

B

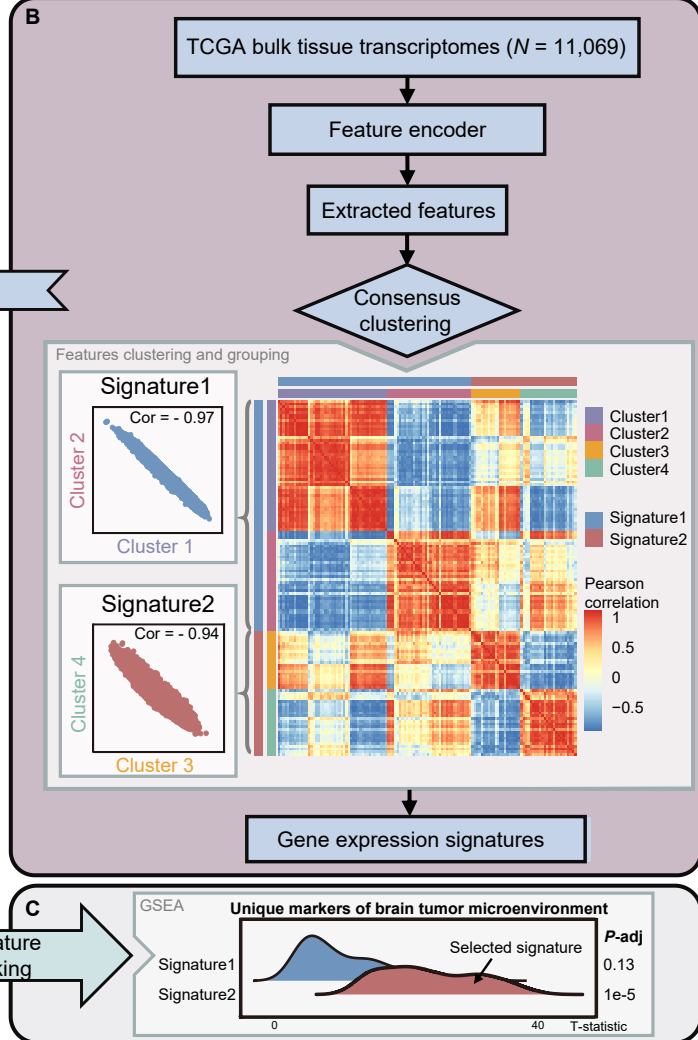

C

Supplement: Supplementary Figure 1 — A perspective of contrastive learning. V0, V0’ are two different views of the same sample X0. The feature encoder represents V0 and V0’ in a reduced dimensional space as R0 and R0’ . Contrastive learning algorithm trains the feature encoder by driving the maximum similarity between R0 and R0’ . [file DataSheet_1.zip › Supplementary material/Figure S3.pdf]

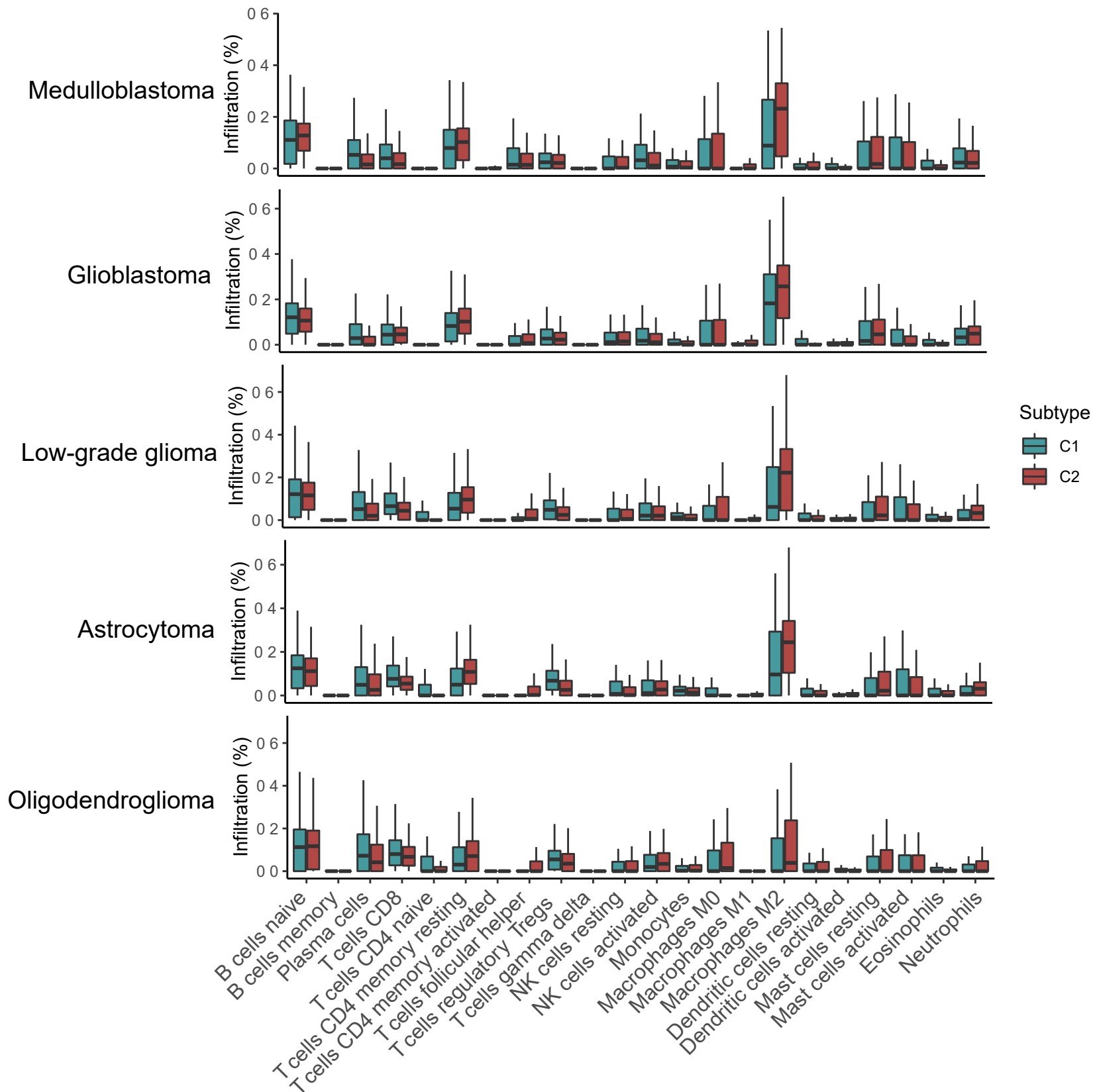

Supplement: Supplementary Figure 1 — A perspective of contrastive learning. V0, V0’ are two different views of the same sample X0. The feature encoder represents V0 and V0’ in a reduced dimensional space as R0 and R0’ . Contrastive learning algorithm trains the feature encoder by driving the maximum similarity between R0 and R0’ . [file DataSheet_1.zip › Supplementary material/Figure S4.pdf]

# Immune signaling gene set

# Hallmark gene set

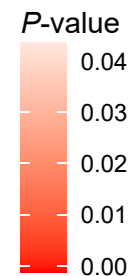

NES (C2 vs. C1)

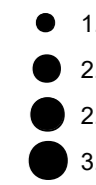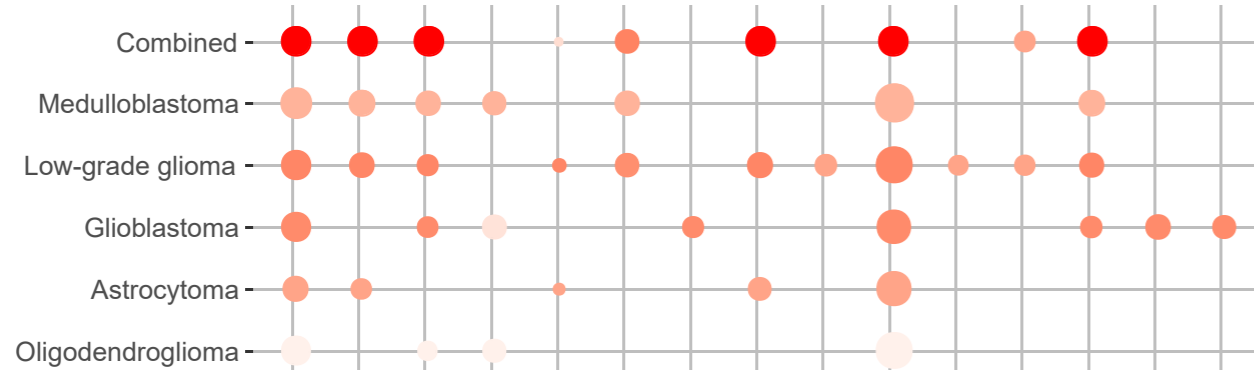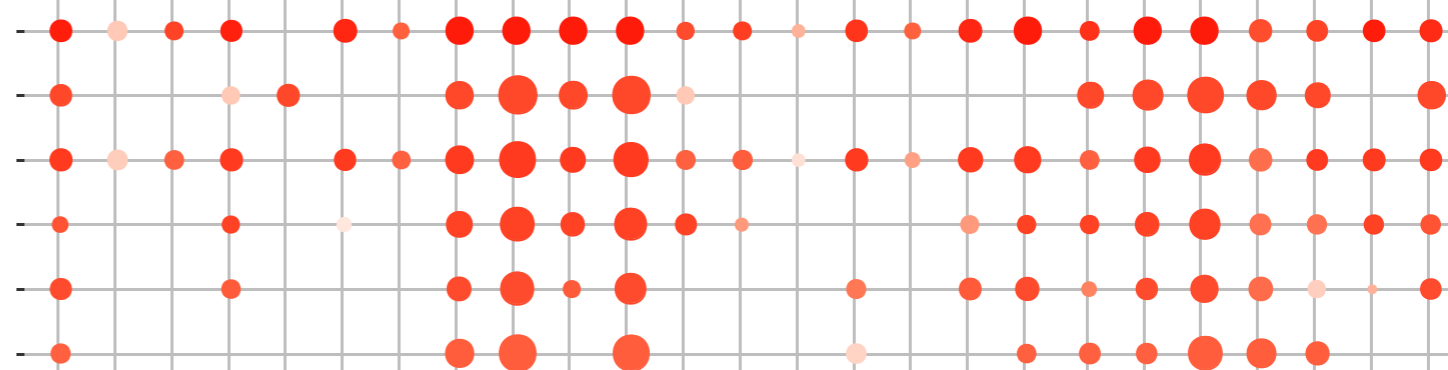

Supplement: Supplementary Figure 1 — A perspective of contrastive learning. V0, V0’ are two different views of the same sample X0. The feature encoder represents V0 and V0’ in a reduced dimensional space as R0 and R0’ . Contrastive learning algorithm trains the feature encoder by driving the maximum similarity between R0 and R0’ . [file DataSheet_1.zip › Supplementary material/Figure S5.pdf]

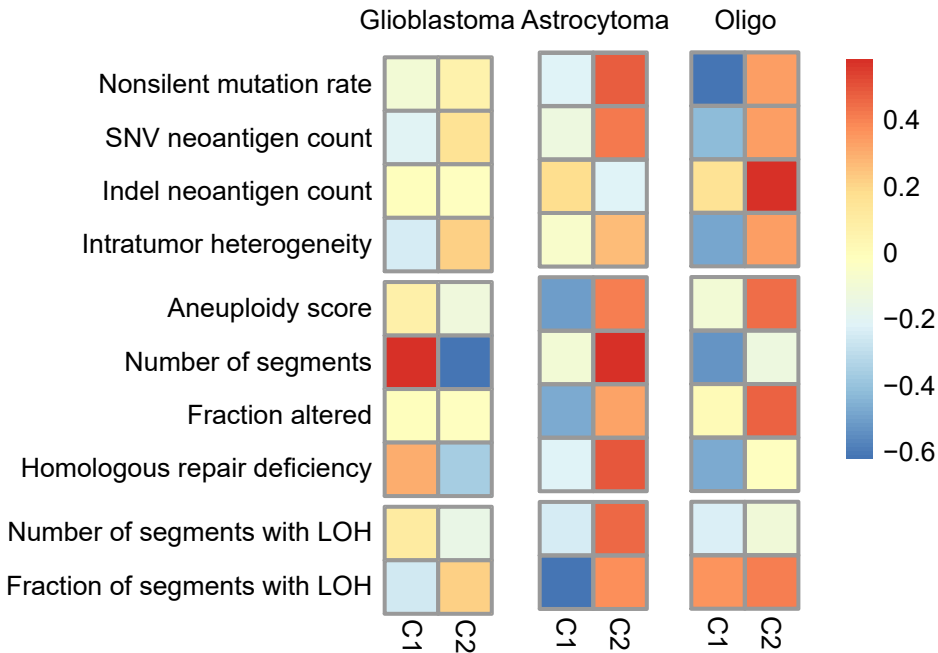

Supplement: Supplementary Figure 1 — A perspective of contrastive learning. V0, V0’ are two different views of the same sample X0. The feature encoder represents V0 and V0’ in a reduced dimensional space as R0 and R0’ . Contrastive learning algorithm trains the feature encoder by driving the maximum similarity between R0 and R0’ . [file DataSheet_1.zip › Supplementary material/Figure S6.pdf]

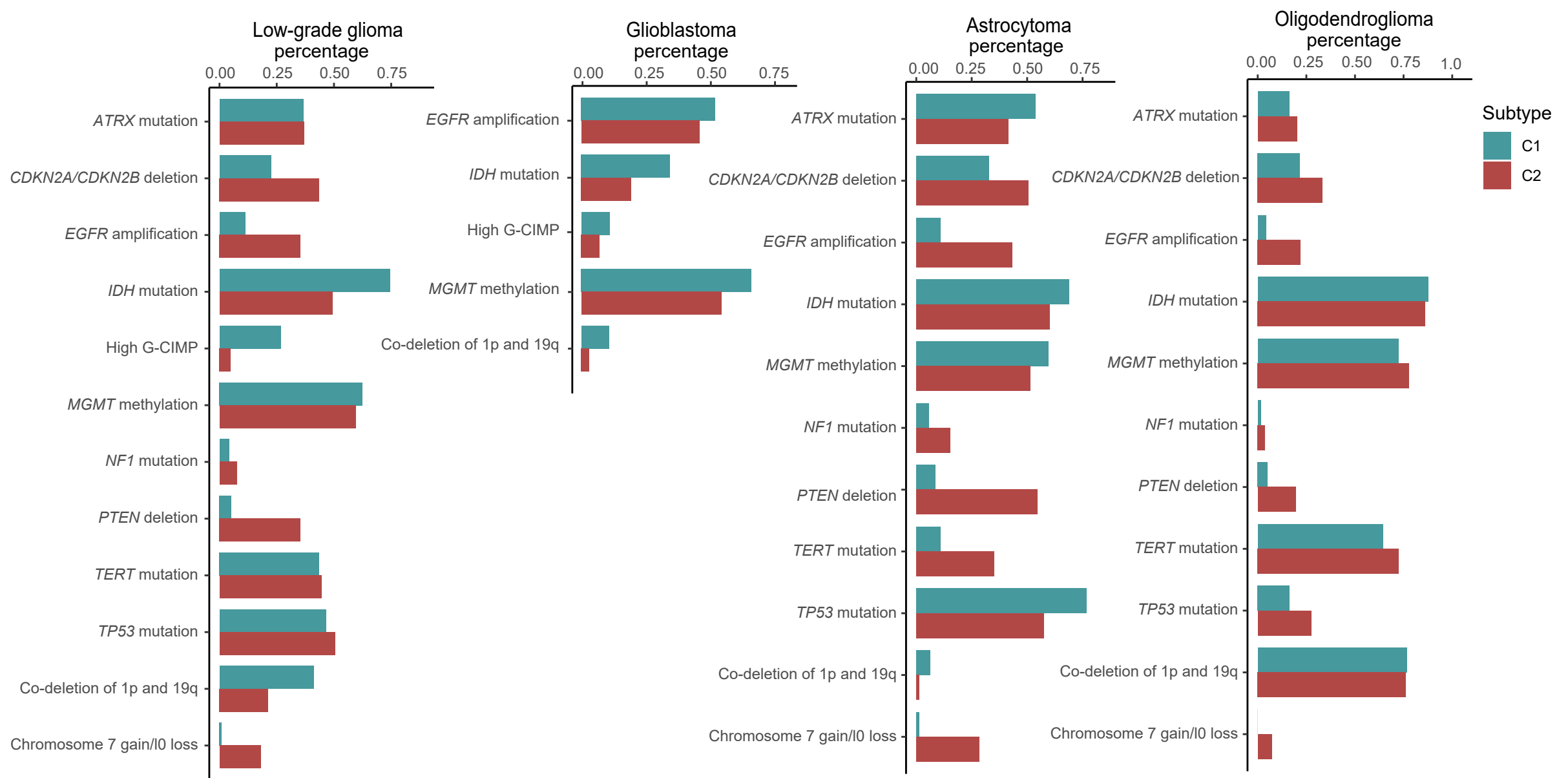

Supplement: Supplementary Figure 1 — A perspective of contrastive learning. V0, V0’ are two different views of the same sample X0. The feature encoder represents V0 and V0’ in a reduced dimensional space as R0 and R0’ . Contrastive learning algorithm trains the feature encoder by driving the maximum similarity between R0 and R0’ . [file DataSheet_1.zip › Supplementary material/Figure S7.pdf]

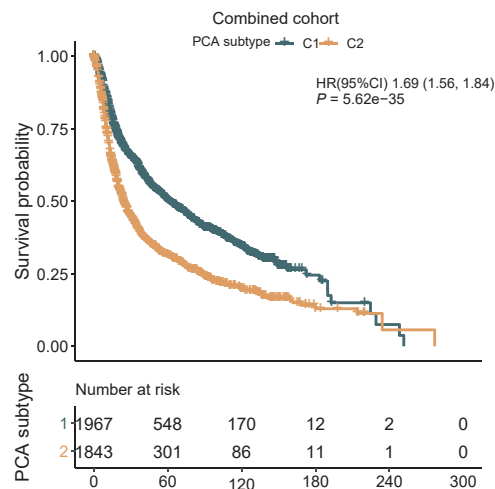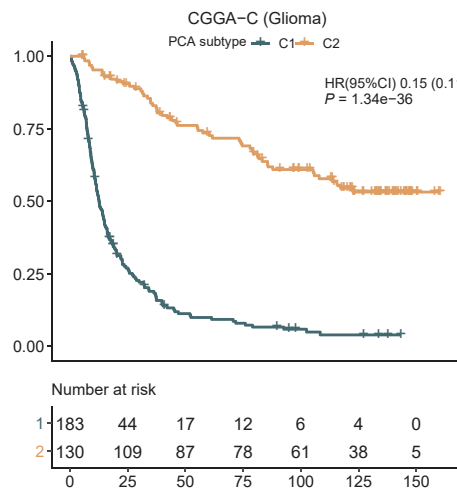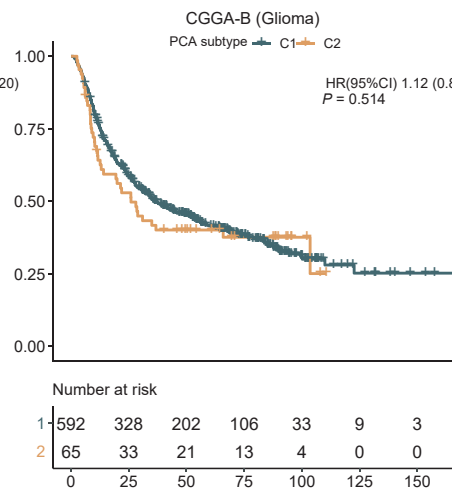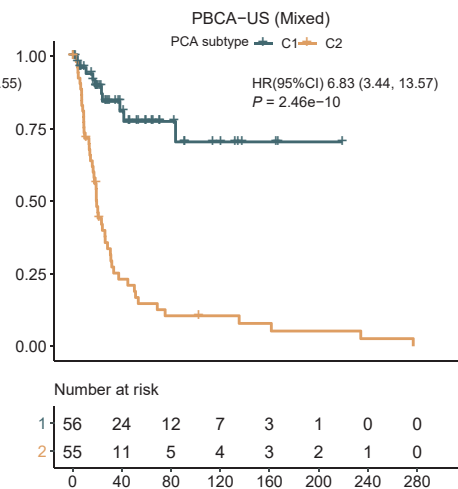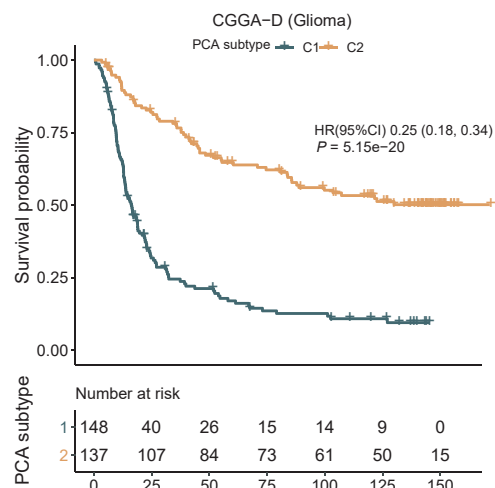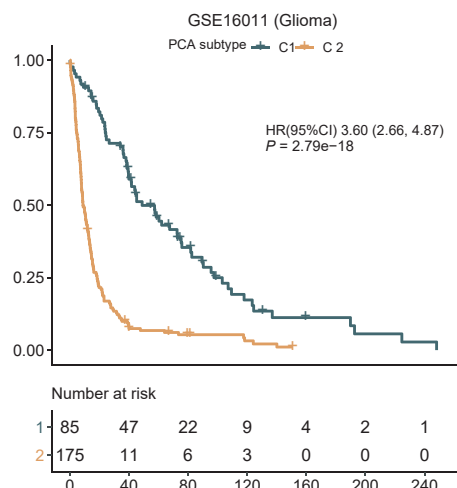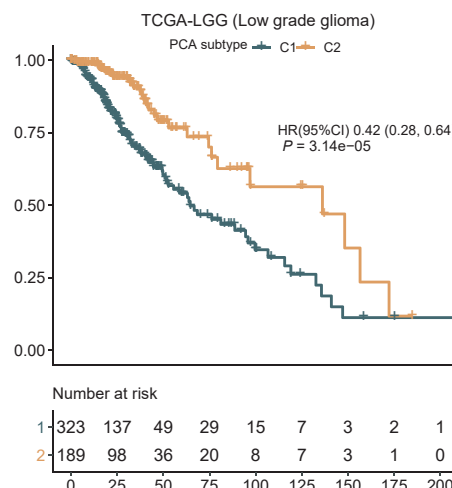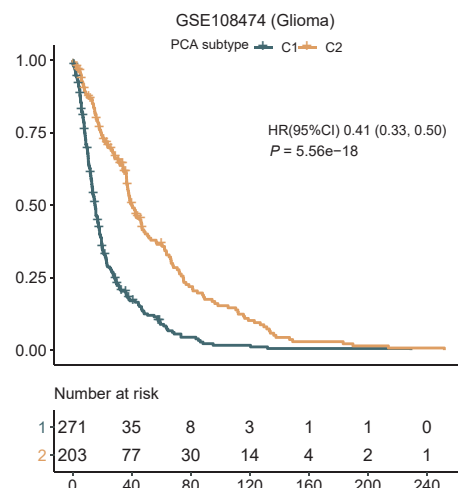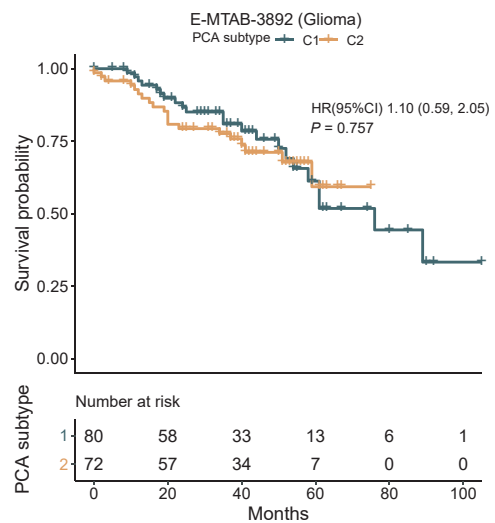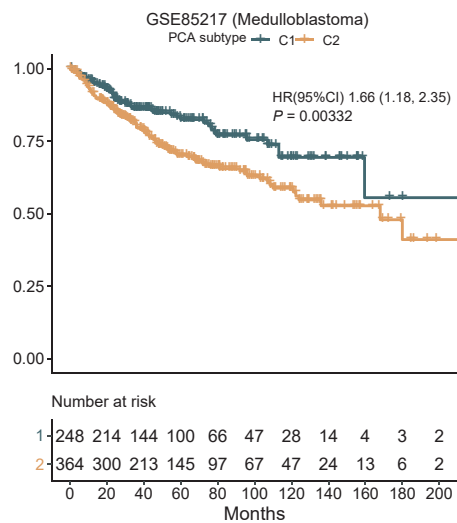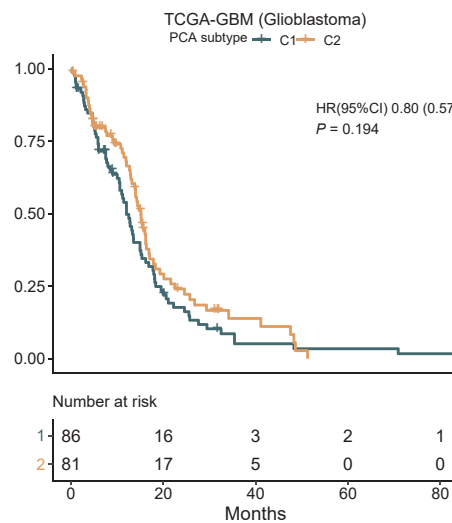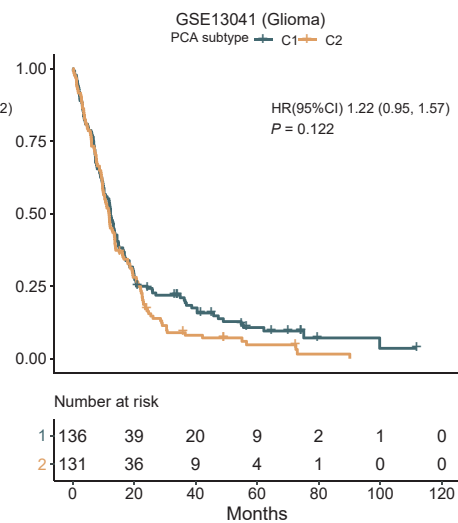

Supplement: Supplementary Figure 1 — A perspective of contrastive learning. V0, V0’ are two different views of the same sample X0. The feature encoder represents V0 and V0’ in a reduced dimensional space as R0 and R0’ . Contrastive learning algorithm trains the feature encoder by driving the maximum similarity between R0 and R0’ . [file DataSheet_1.zip › Supplementary material/Figure S9.pdf]
