# Supplementary material for: Transcriptomic Analysis Identified Two Subtypes of Brain Tumor Characterized by Distinct Immune Infiltration and Prognosis
Source: Front Oncol. 2021 Oct 15;11:734407. doi: 10.3389/fonc.2021.734407 (PMC8554158; doi:10.3389/fonc.2021.734407)
Supplement: Supplementary Figure 1 — A perspective of contrastive learning. V0, V0’ are two different views of the same sample X0. The feature encoder represents V0 and V0’ in a reduced dimensional space as R0 and R0’ . Contrastive learning algorithm trains the feature encoder by driving the maximum similarity between R0 and R0’ . [file Figure S8.pdf › Supplementary material/Figure S8.pdf]

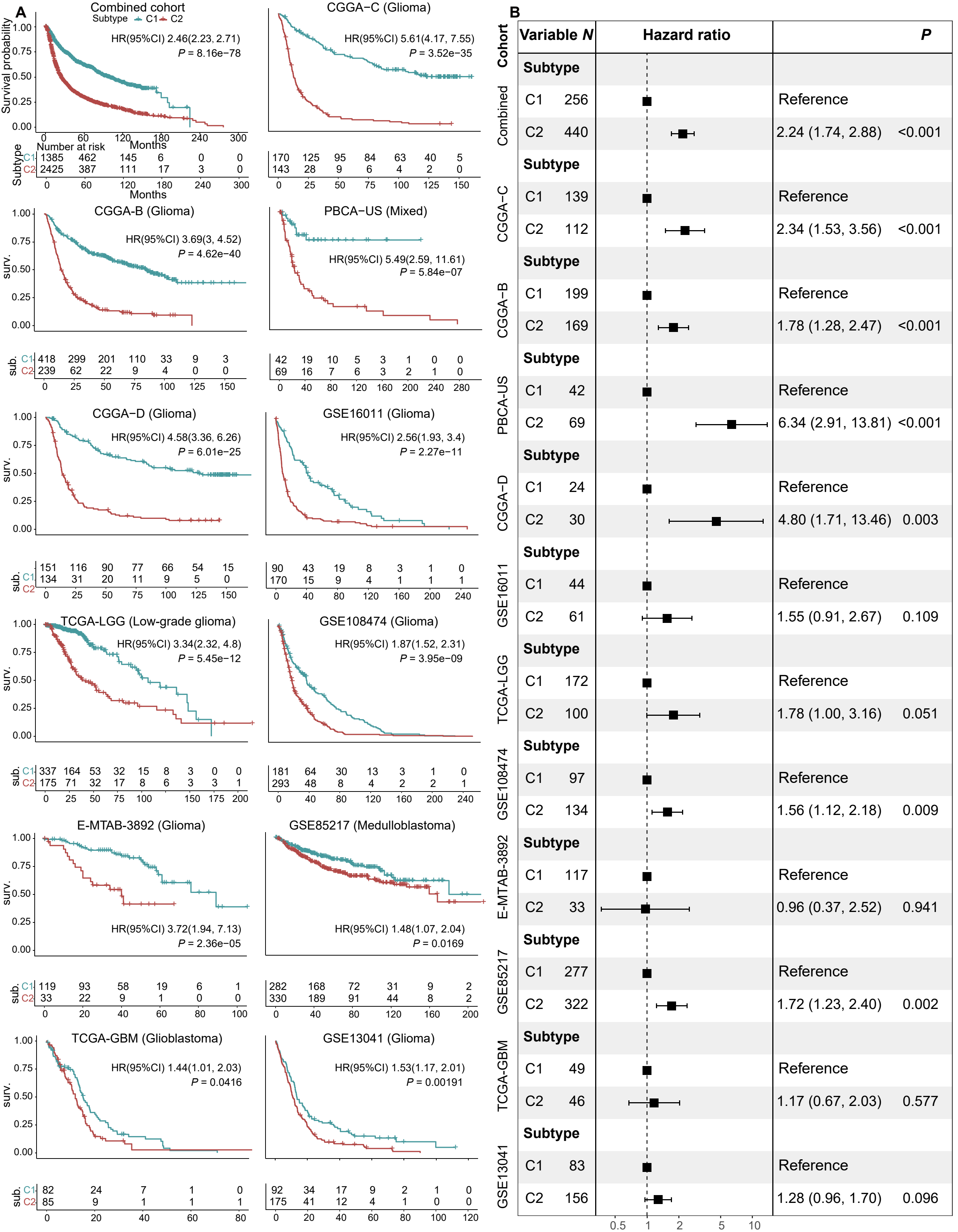
A
Combined cohort
Subtype C1 C2
HR(95%CI) 2.46(2.23, 2.71)
P = 8.16e−78
Survival probability
Months
Number at risk
C1 1385 462 145 6 0 0
C2 2425 387 111 17 3 0
CGGA−C (Glioma)
HR(95%CI) 5.61(4.17, 7.55)
P = 3.52e−35
170 125 95 84 63 40 5
143 28 9 6 4 2 0
CGGA-B (Glioma)
HR(95%CI) 3.69(3, 4.52)
P = 4.62e−40
C1 418 299 201 110 33 9 3
C2 239 62 22 9 4 0 0
PBCA−US (Mixed)
HR(95%CI) 5.49(2.59, 11.61)
P = 5.84e−07
42 19 10 5 3 1 0 0
69 16 7 6 3 2 1 0
CGGA−D (Glioma)
HR(95%CI) 4.58(3.36, 6.26)
P = 6.01e−25
C1 151 116 90 77 66 54 15
C2 134 31 20 11 9 5 0
GSE16011 (Glioma)
HR(95%CI) 2.56(1.93, 3.4)
P = 2.27e−11
90 43 19 8 3 1 0
170 15 9 4 1 1 1
TCGA-LGG (Low-grade glioma)
HR(95%CI) 3.34(2.32, 4.8)
P = 5.45e−12
C1 337 164 53 32 15 8 3 0 0
C2 175 71 32 17 8 6 3 3 1
GSE108474 (Glioma)
HR(95%CI) 1.87(1.52, 2.31)
P = 3.95e−09
181 64 30 13 3 1 0
293 48 8 4 2 2 1
E-MTAB-3892 (Glioma)
HR(95%CI) 3.72(1.94, 7.13)
P = 2.36e−05
C1 119 93 58 19 6 1
C2 33 22 9 1 0 0
GSE85217 (Medulloblastoma)
HR(95%CI) 1.48(1.07, 2.04)
P = 0.0169
282 168 72 31 9 2
330 189 91 44 8 2
TCGA-GBM (Glioblastoma)
HR(95%CI) 1.44(1.01, 2.03)
P = 0.0416
C1 82 24 7 1 0
C2 85 9 1 1 1
GSE13041 (Glioma)
HR(95%CI) 1.53(1.17, 2.01)
P = 0.00191
92 34 17 9 2 1 0
175 41 12 4 1 0 0
B
Cohort Variable N Hazard ratio P
Combined Subtype
C1 256 Reference
C2 440 2.24 (1.74, 2.88) <0.001
CGGA−C Subtype
C1 139 Reference
C2 112 2.34 (1.53, 3.56) <0.001
CGGA−B Subtype
C1 199 Reference
C2 169 1.78 (1.28, 2.47) <0.001
PBCA-US Subtype
C1 42 Reference
C2 69 6.34 (2.91, 13.81) <0.001
CGGA−D Subtype
C1 24 Reference
C2 30 4.80 (1.71, 13.46) 0.003
GSE16011 Subtype
C1 44 Reference
C2 61 1.55 (0.91, 2.67) 0.109
TCGA-LGG Subtype
C1 172 Reference
C2 100 1.78 (1.00, 3.16) 0.051
GSE108474 Subtype
C1 97 Reference
C2 134 1.56 (1.12, 2.18) 0.009
E-MTAB-3892 Subtype
C1 117 Reference
C2 33 0.96 (0.37, 2.52) 0.941
GSE85217 Subtype
C1 277 Reference
C2 322 1.72 (1.23, 2.40) 0.002
TCGA-GBM Subtype
C1 49 Reference
C2 46 1.17 (0.67, 2.03) 0.577
GSE13041 Subtype
C1 83 Reference
C2 156 1.28 (0.96, 1.70) 0.096
0.5 1 2 5 10
